# The Past and Present Lives of the Intraocular Transmembrane Protein CD36

**DOI:** 10.3390/cells12010171

**Published:** 2022-12-31

**Authors:** Rucui Yang, Qingping Liu, Mingzhi Zhang

**Affiliations:** 1 Joint Shantou International Eye Center of Shantou University and The Chinese University of Hong Kong, Shantou 515041, China; 2 Department of Ophthalmology, Shantou University Medical College, Shantou University, Shantou 515041, China

**Keywords:** cluster of differentiation 36, post translational modification, signaling pathway, retina, eye disease, age-related macular degeneration, diabetic retinopathy, glaucoma, intraocular neovascularization, intraocular inflammation

## Abstract

Cluster of differentiation 36 (CD36) belongs to the B2 receptors of the scavenger receptor class B family, which is comprised of single-chain secondary transmembrane glycoproteins. It is present in a variety of cell types, including monocytes, macrophages, microvascular endothelial cells, adipocytes, hepatocytes, platelets, skeletal muscle cells, kidney cells, cardiomyocytes, taste bud cells, and a variety of other cell types. CD36 can be localized on the cell surface, mitochondria, endoplasmic reticulum, and endosomes, playing a role in lipid accumulation, oxidative stress injury, apoptosis, and inflammatory signaling. Recent studies have found that CD36 is expressed in a variety of ocular cells, including retinal pigment epithelium (RPE), retinal microvascular endothelial cells, retinal ganglion cells (RGC), Müller cells, and photoreceptor cells, playing an important role in eye diseases, such as age-related macular degeneration (AMD), diabetic retinopathy (DR), and glaucoma. Therefore, a comprehensive understanding of CD36 function and downstream signaling pathways is of great significance for the prevention and treatment of eye diseases. This article reviews the molecular characteristics, distribution, and function of scavenger receptor CD36 and its role in ophthalmology in order to deepen the understanding of CD36 in eye diseases and provide new ideas for treatment strategies.

## 1. Introduction

In the late 1970s, Goldstein, Brown, and colleagues identified a class of glycoproteins as scavenger receptors, located on the surface of macrophages, that recognize both acetylated low-density lipoprotein and oxidized low-density lipoprotein (ox-LDL) [1,2]. It was later found to be a class of typical pattern recognition receptors involved in innate immunity and lipoprotein metabolism. In 2017, the National Institute of Allergy and Infectious Diseases (NIAID) and National Institutes of Health (NIH) Tissue divided scavenger receptors into 11 families, SR-A to SR-L [3]. Among them was cluster of differentiation 36 (CD36), also known as leukocyte differentiation antigen 36 and fatty acid translocase (FAT), belonging to the B2 receptors of scavenger receptor class B [4], a highly glycosylated single-chain secondary transmembrane protein [5]. CD36 plays an important role in lipid accumulation, inflammatory injury, apoptosis, and oxidative stress [6], and is a core factor in the initiation of atherosclerosis [7,8], diabetes [9], non-alcoholic fatty liver disease [10], and other diseases. In recent years, with the change of lifestyle, the number of people with hypercholesterolemia and obesity caused by overnutrition has gradually increased, and the role of abnormal lipid metabolism in eye diseases has attracted increasingly more attention. CD36 is a well-known fatty acid transporter that plays a role in paper homeostasis and innate immune responses [11]. With the in-depth study of CD36, it has been found that CD36 is involved in the occurrence and development of a variety of human eye diseases. As a key protein regulating lipid metabolism, loss of CD36 can lead to abnormal deposition of lipids in the subretinal area caused by the detachment of the outer segments of retinal photoreceptors [12]. CD36 expression can cause vascular abnormalities in the ocular surface and retina due to its involvement in endothelial dysfunction [13,14]. In addition, as a proximal signal for Toll-like receptor (TLR) 4 and TLR6 accessory receptor [15], CD36 also plays an important role in the intraocular inflammatory response and production of reactive oxygen metabolites (ROM) [16]. Previous studies have found that simvastatin treatment reduces retinal cholesterol and increases the retinal expression of CD36, suggesting that statins may be involved in the treatment of lipid metabolism-related eye diseases through CD36, the main receptor of ox-LDL [17]. Therefore, as a potential therapeutic target, it is important to fully understand the role of CD36 in the eye and the signaling pathways involved in the prevention and treatment of related eye diseases. This paper summarizes the molecular characteristics and functions of CD36 and the physiological and pathological roles of CD36 in eye diseases, especially age-related macular degeneration, diabetic retinopathy, glaucoma, subretinal inflammation, retinal neovascularization, keratitis, and corneal neovascularization, in order to identify CD36 as a therapeutic target for the prevention and treatment related eye diseases.

## 2. Molecular Characteristics of CD36

### 2.1. Structural Features of CD36

The human *CD36* gene is located at locus q11.2 on chromosome 7 and is over 46 kb in length [18], contains 15 exons, is 32 kb long [19].

*CD36* mRNA expression is strictly controlled by two major transcription factors: CCAAT/enhancer-binding protein α (C/EBPα) and C/EBPβ, which can directly bind and regulate the promoter of the *CD36* gene and initiate mRNA transcription. Studies on 3T3-L1 adipocytes, mouse embryonic fibroblasts, and human embryonic kidney 293 cells showed that CD36 protein level is positively correlated with C/EBPα. Interestingly, C/EBPβ functions as a repressor and activator in different cell types; for example, CD36 protein levels were negatively and positively correlated with C/EBPβ in 3T3-L1 adipocytes and mouse embryonic fibroblasts, respectively [20]. Studies have shown that CD36 plays an important role in the development of Alzheimer’s disease-like diseases. Phospho-Smad2/3 can inhibit the phagocytosis of amyloid-beta (Aβ) by microglia and accelerate the progression of Alzheimer’s disease-like diseases by inhibiting the transcription of the *CD36* gene [21]. The E3 ubiquitin ligase Pellino 1 inhibits transcription of the *CD36* gene through C/EBPβ [22]. In contrast, triggering receptor expressed on myeloid cells 2 can regulate C/EBPα-dependent CD36 expression and subsequent Aβ phagocytosis to prevent Alzheimer’s disease. This process may be related to Akt phosphorylation [23]. Peroxisome-proliferator-activated receptor (PPAR)γ is a nuclear receptor transcription factor that can regulate adipogenesis and energy homeostasis. PPARγ is one of the transcription factors for *CD36*. Studies have shown that PPARγ is involved in the expression of CD36 in macrophages [24], cardiac microvascular endothelial cells [25], and human HK-2 proximal tubular cell lines [26]. The transcription complex formed by retinoic acid X receptor and PPARγ can activate CD36 transcription.

CD36 protein is composed of 472 amino acids, and its presumed relative molecular mass is 53 kDa. However, the actual mass of this protein varies from 78 to 88 kDa [19], depending on the cell type and extent of glycosylation [27]. CD36 after complete glycosylation is about 88 kDa [6]. There are ten potential glycosylation sites in the extracellular region of rat CD36, eight of which are conserved between humans and rats [28].

### 2.2. Posttranslational Modifications of CD36

Posttranslational modifications, including ubiquitination, glycosylation, phosphorylation, and palmitoylation, may have important effects on the synthesis, distribution, and function of CD36 (protein stability, protein folding, transport, and ligand uptake rate). It is worth noting that the function of CD36 acetylation still needs to be further studied.

**Figure 1:**
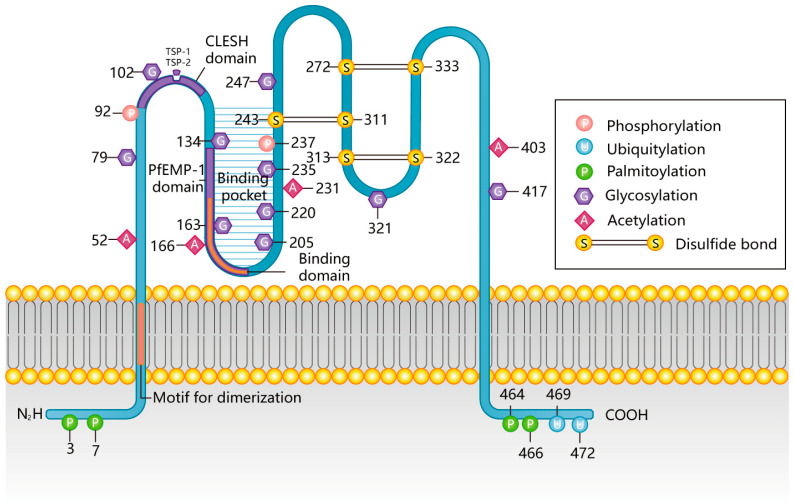
Protein structure of CD36.

CD36 is mainly divided into five regions: the carboxy-terminal intracellular domain (COOH-terminal), the amino-terminal intracellular domain (NH2-terminal), an extracellular domain, and two transmembrane domains. The C and N termini of CD36 are intracellular, and both ends are palmitoylated (3/7, 464/466). In addition to palmitoylation, the COOH terminus also contains a pair of ubiquitination sites (469/472) [6,29] and has a motif of CXCX5K located on the cytosolic ends of the T cell co-receptors CD4 and CD8, which may be involved in the binding of src-related protein tyrosine kinases [30]. This latter region is considered to be the main binding site for CD36, which binds intracellular signaling molecules and mediates signaling downstream of CD36 [18]. The extracellular domain, recognized by the ligand, is a highly glycosylated hydrophobic ring containing 10 glycosylation sites (79/102/134/163/205/220/235/247/321/417), two phosphorylation sites (92, 237), and three pairs of disulfide bonds (243-311/313-322/272-333) [6,31,32,33]. The specific sites of post-translational modification of CD36 in and out of cells and the binding region of CD36 with the ligand are shown in Figure 1 [6]. These extracellular sites not only undergo post-translational modification but also interact with a series of ligands to recognize foreign microorganisms and diseased cells in vivo.

#### 2.2.1. CD36 Palmitoylation

Palmitoylation occurs in the endoplasmic reticulum [31], where palmitoylated CD36 is located in lipid rafts of cell membranes and is involved in subcellular transport, localization of proteins, and absorption of fatty acids [32]. Studies have shown that inhibition of CD36 palmitoylation can promote the localization of CD36 to hepatocyte mitochondria to alleviate nonalcoholic fatty liver disease [33]. Palmitoylation occurring at the amino and carboxyl cysteine residues of CD36 is reversible and requires palmitoyl transferases and palmitoyl protein thioesterases for palmitoylation and depalmitoylation, respectively [34].

Selenoprotein K may also be involved in the palmitoylation of CD36 [35]. Plasma ceruloplasmin [31] and insulin [36] inhibit and enhance palmitoylation, respectively. Ox-LDL has recently been found to increase CD36 palmitoylation [37]. When the palmitoylation of CD36 is inhibited by plasma ceruloplasmin, CD36 is processed in the ER and its transport through the secretory pathway was significantly prolonged, and results in the CD36 precursor protein being retained in the ER [31]. No studies have revealed the role of CD36 palmitoylation in eye disease.

#### 2.2.2. CD36 Ubiquitination

Ubiquitination of CD36 is mainly mediated by E3 ubiquitin ligase [38]. Whether or not ubiquitination and the degree of ubiquitination affects the stability and degradation of protein, it does not participate in the subcellular localization and transport of the protein [39]. As a ligand for CD36, long-chain fatty acids (LCFA) mediate polyubiquitin degradation of CD36 and reduce fatty acid uptake [34,39]. In addition, platelet-derived exosomes appear to downregulate CD36 protein expression through similar ubiquitination-related functions. Parkin is an E3 ubiquitin ligase that mediates polyubiquitination of CD36 in intestinal epithelial cells to reduce CD36 protein levels [40]. Interestingly, in addition to mediating polyubiquitination, Parkin also mediates CD36 mono-ubiquitination to stabilize protein structure, increase protein levels, form protein complexes, and increase fatty acid uptake and lipid accumulation [41]. Ubiquitin-specific peptidase (USP) 10 [42], USP14 [43], ubiquitin C-terminal hydrolase (UCHL)1 [44], and insulin [39] are involved in the deubiquitination of CD36, which can prevent the CD36 protein from being transported to the proteasome for degradation, stabilize the protein, and maintain insulin signal transduction. USP10 can interact with CD36 by removing the polyubiquitin on CD36 to stabilize the CD36 protein, thereby promoting foam cell formation and lipid accumulation and promoting the development of atherosclerosis, whereas inhibition or knockdown of USP10 can have the opposite effect [42]. As a deubiquitinase, inhibition of UCHL1 inhibits lipid uptake by increasing the abundance of K48-polyubiquitin on CD36 and thereby blocking its activation, thus suggesting that UCHL1 may be a potential target for atherosclerotic therapy [44], which may provide a new insight into the protein degradation of CD36 in lipid-metabolism-related ophthalmic diseases with similar pathogenesis to atherosclerosis.

#### 2.2.3. CD36 Glycosylation

Glycosylation of CD36 occurs in the endoplasmic reticulum and Golgi apparatus and is involved in the regulation of the formation of the correct spatial structure of CD36, protein stability, and cell membrane localization, and is therefore essential for CD36 expression, transport, and fatty acid absorption [45]. However, ligand recognition, such as CD36 binding to modified low-density lipoprotein (LDL), is not affected by altered glycosylation patterns [46]. In vivo experiments show that CD36 protein level is downregulated and fatty acid intake is decreased in spontaneous hypertensive rats induced by a mutation at Asn102. Therefore, the mutation of *CD36* at the Asn102 glycosylation site may affect the structural stability and cell membrane localization of CD36, thereby affecting protein expression and fatty acid absorption, but the specific mechanism needs to be further confirmed [47]. As the only sugar modification of proteins present in the cytoplasm and nucleus, O-GlcNAcylation is associated with protein localization and functional regulation. Recent studies have shown that high O-GlcNAcylation induced by a high-fat diet promotes transcription and activity of CD36 through activation of the NF-κB pathway and direct modification of CD36 at Ser468 and Thr470, thereby driving gastric cancer metastasis [48]. Studies have shown that mutations at Asn108 and Asn173 lead to abnormal distribution of CD36 on COS M6 cell membranes [49]. Studies have demonstrated that the carboxyl terminal Asn247, Asn321, and Asn417 are indispensable for CD36 transport [46], and that glucosamine O-GlcNAc transferase (OGT) in perfused heart mediates the connection between O-linked N-acetylglucosamine (O-GlcNAc) and CD36, induces CD36 translocation to the sarcolemma, and increases CD36 abundance [50]. This is followed by increased uptake of fatty acids by cardiomyocytes. Clinical studies in patients with liver cirrhosis have also confirmed that CD36 glycosylation may promote hepatic uptake of LCFA, which may be part of the pathogenesis of liver inflammation and liver cancer [51], and this may provide a new idea for the treatment of eye diseases associated with long-chain fatty acids by dietary regulation. 

#### 2.2.4. CD36 Phosphorylation

Thr92 and Ser237 of CD36 are phosphorylated by protein kinase C and protein kinase A [52,53], respectively, and can be dephosphorylated by intestinal alkaline phosphatase in small intestinal epithelial cells [54]. Phosphorylation of CD36 at Thr92 can inhibit the binding of platelets to thrombospondin (TSP)-1 and increase platelet adhesion to collagen [55]. However, the dephosphorylation of Thr92 of CD36 protein has the opposite effect on the rolling and adhesion of Plasmodium falciparum-infected erythrocytes to human dermal microvascular endothelial cells [55]. Phosphorylated CD36 at Ser237 downregulates fatty acid uptake in platelets and intestinal epithelial cells [56]. However, it has been suggested that Thr92 phosphorylation does not block the interaction directly, as no evidence of Thr92 phosphorylation was seen in the electron density map [5], and thus Thr92 phosphorylation needs to be further investigated. Phosphorylated CD36 at Ser237 downregulates fatty acid uptake in platelets [56]. In vitro studies showed that G protein-coupled receptor kinase-2 directly phosphorylates CD36, although the specific site of action is unclear [57]. There have been few studies on CD36 phosphorylation in recent years. Whether CD36 phosphorylation also affects the retinal uptake of fatty acids has not been investigated. 

#### 2.2.5. CD36 Acetylation

Proteomic and mass spectrometry analysis showed that the CD36 acetylation sites in rats and humans are identical and are located at Lys52, Lys166, Lys231, and Lys403 [27,58,59]. The lysine deacetylase inhibitors trichostatin A or valproic acid, by affecting the acetylation-deacetylation signal transduction, regulate CD36 function and subsequent lipid accumulation in pancreatic β-cells exposed to glucolipotoxicity [60]. Acetylation itself is relatively little studied, and the specific function of acetylation in the retina still needs to be further studied.

### 2.3. CD36 Distribution

Posttranslational modification may be the main determinant of CD36 cell location and function. CD36 protein exists in monocytes, macrophages, microglia, microvascular endothelial cells, adipocytes, hepatocytes, platelets, skeletal muscle cells, kidney cells, cardiomyocytes, taste bud cells, and many other cell types [27,29,61,62]. Moreover, it has been linked to many diseases, including atherosclerosis, diabetes, cardiovascular disease, cancer, and Alzheimer’s disease.

In cells, CD36 is found on the cell surface, in mitochondria, the endoplasmic reticulum, Golgi apparatus, and endosomes [63]. CD36 protein is synthesized in polyribosomes, further processed in the endoplasmic reticulum and Golgi apparatus, and then transported to the cell membrane by endosomes. CD36 is also found in mitochondria, although its exact function is unclear. Elevated insulin [64] levels and muscle contraction [65] stimulate PI3K-AKT signaling and adenosine monophosphate kinase, respectively. Ultimately, both promote the net translocation of CD36 to the plasma membrane through the guanosine triphosphate/guanosine diphosphate cycle for different functions. Posttranslational modifications, especially palmitoylation and glycosylation, may play an important role in the intracellular localization of CD36.

Recent studies have found that CD36 is expressed in a variety of ocular cells. Houssier et al. showed that CD36 is mainly located on the basal surface of rat retinal pigment epithelial cells and retinal microvascular endothelial cells [66]. Tserentsoodol et al. localized CD36 in monkey retina by the immunofluorescence method and found that CD36 not only existed in the RPE layer but also in the ganglion cell layer and Müller cell layer, and the outer plexiform layer was also labeled. Meanwhile, the inner segments of rod photoreceptors are strongly labeled, but the inner segments of cone photoreceptors are not. RPE showed punctate labeling different from lipofuscin particles [67]. In addition, the apex of the outer segment is brightly marked, consistent with the known role of this receptor in the rod-shaped outer segment phagocytosis by RPE [68]. Yoon et al. showed that CD36 exists not only in the retina but also in the cornea and conjunctiva regions, and there was no significant difference in the expression of CD36 between young and old donor eyes [69,70].

### 2.4. CD36 Ligands

The ligands that CD36 protein can bind mainly include three types: (1) A lipid-related ligand such as LCFA [71], ox-LDL [72,73], and oxidized phospholipid (ox-PL) [74]. (2) Protein-associated ligands: advanced oxidation protein products [75], advanced glycation end products [76], TSP-1 [77], TSP-2 [78], S100 family proteins (S100-A8, S100-A9 [79], S100-A12 [80]), amyloid [81], and synthetic-growth-hormone-releasing peptide family members (hexarelin [82], EP 80317 [83]). (3) Foreign microorganisms and diseased cells in the body: Plasmodium falciparum infects red blood cells, bacterial cell wall components of Staphylococcus and Mycobacterium, cell-derived particles, and apoptotic cells [84,85]. The ligand recognition region of CD36 mainly includes three segments:

1. CD36, limP-2, Emp sequence homology (CLESH) binding sites (93–120 and 155–183): CLESH is a 30-residue, long, negatively charged domain in CD36 that interacts with thrombospondin structural homology repeat with a positively charged surface ridge with high affinity. After binding, macrophages initiate the binding and entrainment of apoptotic neutrophils to produce IL-10 [78,86,87,88].
2. *P. falciparum* erythrocyte membrane protein-1 (PfEMP-1)-binding site (139–184, 146-164AA, or 145-171AA, to be exact): This region binds PfEMP-1, a membrane protein specifically expressed by erythrocytes infected with Plasmodium falciparum [89].
3. Lipid and protein binding site (155–183): This region contains a positive groove formed by a lysine cluster and hydrophobic amino acids that can recognize bound lipids such as ox-LDL (155–183) [90], oxidized phospholipids (157–171) [85], and long-chain fatty acids (127–279) [91]. In addition, this region can bind proteins such as advanced glycosylation end products [92] and members of the synthetic-growth-hormone-releasing peptide families such as hexarelin and EP 80317 [93]. Other possible ox-LDL binding sites on CD36 are 28–93 and 120–155 [94].

Many of these ligands can cause pathological damage such as abnormal lipid metabolism, oxidative stress injury, apoptosis, and inflammation, playing an important role in the occurrence and development of eye diseases such as age-related macular degeneration, diabetic retinopathy, retinal ganglion cell injury, and intraocular neovascularization. The roles of CD36 in different cell types and eye diseases are shown in Table 1.

Studies have identified definitive CD36 agonists and inhibitors. KDdiA-PC, a CD36 agonist, is a ligand for ox-LDL and the macrophage scavenger receptor CD36 [110]. Sulfosuccinimidyl oleate sodium (Sulfo-N-succinimidyl oleate sodium) is a long-chain fatty acid that irreversibly inhibits the transport of fatty acids to cells and is also an effective and irreversible inhibitor of the mitochondrial respiratory chain. Sulfo-N-succinimidyl oleate sodium binds to the CD36 receptor on the surface of microglia and has anti-inflammatory effects [111], having been shown to inhibit the role of islet CD36 in FA uptake [112]. 6-Thioinosine, a purine antimetabolite, is an antiadipogenic agent that can reduce the mRNA levels of PPARγ and C/EBPα and its target gene CD36 [113].

## 3. The Function of CD36

The CD36 protein is synthesized in polyribosomes, further processed, and synthesized in endoplasmic reticulum and Golgi apparatus, and then following this is transported to the cell membrane by endosomes. CD36 is also distributed in mitochondria, but its specific function remains unclear. Posttranslational modifications, especially palmitoylation and glycosylation, may play an important role in the intracellular localization of CD36.

The CD36 protein exists in monocytes, macrophages, microglia, microvascular endothelial cells, adipocytes, hepatocytes, platelets, skeletal muscle cells, kidney cells, cardiomyocytes, and many other cell types [27,29,61,62]. Houssier and Tserentsoodol et al. showed that CD36 was mainly expressed in the basal surface of rat retinal pigment epithelial cells, Müller cells, and the inner segments of photoreceptor cells, and could also be localized in retinal microvascular endothelial cells and ganglion cells [66,67]. Yoon et al. showed that CD36 exists not only in the retina but also in the cornea and conjunctiva regions [69,70].

CD36 can recognize and bind a variety of endogenous or exogenous ligands; play a variety of biological functions in lipid accumulation, apoptosis, inflammatory signaling, and oxidative stress injury; and have different biological effects.

### 3.1. Mediation of Lipid Recognition and Intake

CD36, also known as FAT, is considered to be the main receptor for the recognition and transport of fatty acids and other lipids in the human body [33]. Palmitoylated CD36 mediates the endocytosis of lipids through lipid valves on the surface of cell membranes [32]. CD36 on macrophages can recognize endogenous lipid ligands such as oxidized phospholipids and ox-LDL to participate in their phagocytosis and transport [109] and then participate in lipid metabolism. The main binding sites of CD36 and ox-LDL are 157–171 [90]. LCFA is an important substrate for human production capacity [114]. CD36 in adipocytes, skeletal muscle cells, cardiomyocytes, and hepatocytes can recognize exogenous LCFA and transport it into cells to provide energy for cell growth and development [115]. A large number of studies have shown that excessive ox-LDL and LCFA can affect normal fatty acid metabolism, cause the accumulation of intracellular lipids and the formation of foam cells, and participate in the occurrence and development of atherosclerosis [18]. RPE cells in the retina have lipid phagocytosis similar to macrophages [95], and thus targeting FAT/CD36-mediated lipid transport may be an effective strategy for treating eye diseases related to lipid metabolism.

### 3.2. Involved in Inflammation

As a typical pattern recognition receptor, CD36 can recognize some pathogen-associated molecular pattern ligands and can also directly recognize endogenous ligands such as ox-LDL [73] and apoptotic and necrotic cells [116], triggering immune responses to remove pathogens in time. It also initiates and regulates immune defense function. As a co-receptor of TLR, CD36 can also bind with TLR4 and TLR6 to form a novel heterotrimeric complex, activate the nuclear factor NF-κB pathway, release inflammatory factors such as interleukin-1β (IL-1β) and tumor necrosis factor-α (TNF-α), and thus participate in inflammatory reaction [100].

The study of Muller and Rhoads et al. found that proinflammatory M1 macrophages and anti-inflammatory M2 macrophages were in a state of balance in vivo, and the abnormal increase in CD36 could break their equilibrium state and lead to polarization imbalance. The nucleotide-binding oligomerization dome-like receptor protein 3 (NLRP3) inflammasome may be involved [117,118].

### 3.3. Regulation of Apoptosis and Angiogenesis

The thrombospondin (TSP) family contains multiple members such as TSP-1 and TSP-2 that belong to extracellular matrix proteins, among which TSP-1 has been more reported in the progression of ophthalmic diseases, has multiple domains, and interacts with many cell surface receptors in vertebrates [119]. Jiménez et al. showed that CD36, as a receptor for TSP-1 and related proteins in microvascular endothelial cells, was involved in the blocking effect of TSP-1 on angiogenesis. Activation of subsequent downstream signaling pathways includes Src family kinases p59Fyn, caspase-3-like proteases, and p38 mitogen-activated protein kinases (MAPKs) [120]. Therefore, TSP-1 can interact with CD36 to regulate cytoskeletal organization, adhesion, migration, and apoptosis; limit blood vessel density in normal tissues; and block the generation and development of pathological blood vessels, being a naturally occurring angiogenesis inhibitor leading to apoptosis [99].

## 4. CD36 with Eye Diseases and Pathological Changes

### 4.1. Fundus Diseases and Pathological Changes

#### 4.1.1. CD36 and Age-Related Macular Degeneration (AMD)

Age-related macular degeneration (AMD) is a complex eye disease that often causes irreversible blindness in the elderly. The degeneration of RPE, choroidal capillaries, and photoreceptor cell death were the main manifestations [121].

Phagocytic cells are mainly divided into specialized and non-specialized types, which distinguish living cells and designated cell materials such as living cell fragments and apoptotic cells by receptors such as MerTK and CD36 expressed on the cell membrane [122,123]. Unlike the previous two, the specialized phagocyte type is a relatively new phagocyte type that includes RPE and Sertoli cells [122]. Although specialized phagocytes and non-specialized phagocytes are epithelial-derived stromal cells, they have functions such as glucose and cholesterol transport, barrier, support, and immune regulation [124]. Phagocytic RPE is a polarized monolayer of epithelial cells that performs many photoreceptor health maintenance functions and participates in the absorption, transport, and degradation of photoreceptor outer segments (POS) [11,12]. The POS located at the tip of the photoreceptor is composed of a multi-layer phospholipid bilayer, which are shed with circadian rhythm and are bound, recognized, and phagocytosed by the apical end of RPE cells with phagocytosis function in order to carry out effective metabolism to remove photooxidative wastes accumulated in the process of light transduction [125]. This process occurs mainly in the morning, and each RPE cell serves about 25 POS, making it arguably the busiest macrophage in the body [95]. In conclusion, RPE and photoreceptors play a synergic role in the maintenance of photoconduction homeostasis. Studies have shown that oxidative damage of RPE leads to dysfunction, which is a key component in the pathogenesis of AMD and may promote the release of extracellular vesicles (EVs) from RPE [11,97]. If POS phagocytosis is not rhythmic, there is a late-onset cumulative phenotype characterized by visual loss and lipofuscin accumulation, which is typical of AMD [96].

The receptor-mediated phagocytosis of POS by RPE is divided into two independent steps. Firstly, the integrin αvβ5 receptor initiates the signaling pathway [96], and secondly, MerTK activates the POS internalization mechanism [98]. Studies from rat (RPE-J) or human (ARPE-19) stable RPE cell lines found that CD36 expression is stable [95]. Blocking CD36 by CD36 antibody or anionic phospholipid can partially inhibit the uptake of POS by RPE in vitro [126]. CD36 as a phosphatidylserine (PtdSer/PS) receptor is sufficient to confer the ability of non-macrophage RPE to phagocytose apoptotic cells to participate in the elimination of POS [127].

The secondary transmembrane protein CD36 is associated with lipid rafts in macrophages, and the finding that scavenger receptors, including CD36, localized and/or migrated in whole or part during POS phagocytosis by RPE, confirmed that RPE cells have a similar situation to macrophages [95]. Some studies have shown that CD36 is involved in POS binding process but not internalization [12]. Zhao et al. suggested that RPE cells have a double function of engulfing rod outer segment membranes and fibronectin, while rod outer segment membranes lack a competitive effect on fibronectin. This indicates that RPE uses different receptors in the phagocytosis of the two. Phagocytosis of rod outer segment may include CD36 and αVβ5 [128], and phagocytosis of fibronectin is mainly mediated by α5β1 integrin. Phagocytosis of fibronectin is mainly mediated by α5β1 integrin [129]. Many studies have also shown that CD36 does not participate in the αVβ5 integrin-dependent phase of RPE phagocytosis, acts independently of αVβ5, and only participates in POS internalization [130,131]. Thus, CD36 may function primarily as a signaling molecule. Roggia et al. upregulated peroxisome-proliferator-activated receptor γ coactivator-1α (PGC-1α) by siRNA and blocking antibodies of CD36 and MerTK, while αVβ5 integrin siRNA and FAK inhibitors inhibited PGC-1α upregulation [12]. Thus, it was proved that αVβ5 integrin and FAK, not CD36, upregulated PGC-1α. Through αVβ5 integrin /FAK/PGC-1α pathway, it alleviated choroidal capillary dysfunction, lysosome accumulation, and Bruch’s membrane (BM) thickening, thereby protecting RPE.

Chang et al. used immunoprecipitation and antibody inhibition experiments to show that it is the interaction of CD81 on RPE rather than CD9, Mer tyrosine kinase, or CD36 with αVβ5 integrin that regulates the availability of αVβ5 integrin binding to POS particles and maintains metabolic stability by changing the activity of αVβ5 receptor [132], participating in the first step of POS engulfing by RPE. Therefore, POS phagocytosis by RPE requires both αVβ5 and CD81, independent of CD36.

POS membrane phospholipid content of close to 25%, mainly composed of neutral phospholipid of phosphatidyl choline (PC) and phosphatidyl ethanolamine (PE), accounted for about 80%. Negatively charged PS and phosphatidylinositol (PI) accounted for 13% and 7%, respectively [126]. In pathological cases, abnormal external exposure of PS and PI may be specifically recognized and phagocytosed by circulating monocytes or macrophages [133].

Oxidized phosphatidylcholines (oxPCs), a high-affinity ligand for CD36 (oxPC_CD36_), selectively inhibits ox-LDL binding to CD36 transfected cells. OxPC_CD36_ produced by oxidation is widely present in atherosclerotic plaques [134]. Sun et al. found that oxidative stress induced by intense light in the dark-adapted rat retina “oxidized” phosphatidylcholine in the outer segment of photoreceptors, producing a new structure-specific oxidized phosphatidylcholine molecule oxPC_CD36_ from 1-palmitoyl-2-linoleyl-*sn*-glycerol-3-phosphatidylcholine (PLPC), 1-palmitoic-2-arachidonyl-sn-glycerol-3-phosphatidylcholine (PAPC), and the docosahexaenoate ester of 2-lysophosphatidylcholine (DHA-PC) [133]. CD36 knockdown mice were used to demonstrate that RPE-mediated POS internalization was achieved through a specific interaction between CD36 and oxPC_CD36_ [133]. Experiments by Ryeom and Sparrow et al. showed that it was PS, and PI liposomes but not PE that competitively bound RPE with purified POS and was internalized. However, no binding of RPE to PS or PI was found in the RPE of mutant rats without CD36 expression. Therefore, oxPCs, PS, and PI may be physiological signals for CD36 to recognize POS on RPE [126].

Using human retinal epithelial cells (ARPE19) in vitro, Gordiyenko et al. revealed that RPE cells internalize ox-LDL via CD36 [135]. CD36 of RPE cells in AMD may internalize oxidized form of cholesterol through LDL, leading to subretinal lipid deposition and accumulation of ox-LDL at the basal side of RPE or BM level, causing lipid metabolism disorders and producing the drusen characteristic of AMD. This causative factor in the membrane region of RPE-Bruch is similar to the cytotoxicity of ox-LDL accumulation in the atherosclerotic machinery [135]. Studies have shown that the phagocytic capacity of CD36 seems to be enhanced under the conditions of lipid stress or oxidative POS in AMD eyes [136]. Studies on macrophages have also shown that macrophages have a higher binding capacity to oxidative PtdSer [137]. The conclusions of these two studies are consistent.

Houssier et al. suggested that CD36 deficiency downregulates retinal POS-induced proangiogenic cyclooxygenase (COX)-2 and vascular endothelial growth factor (VEGF) expression, resulting in choroidal capillary rarefacialization and photoreceptor and choroidal degeneration, leading to dry AMD [66]. As an important participant in POS phagocytosis by RPE, CD36 receptor density affects phagocytosis kinetics, which can be used as one of the criteria for the judgment of cell phagocytosis. Westenskow et al. demonstrated that RPE from the induced pluripotent stem (IPS-RPE) had a good phagocytosis function for POS by flow cytometry detection of αVβ5 integrin, CD36, MerTK receptor expression density, and binding and internalization kinetics at different differentiation stages of RPE. It may be a good substitute for diseased RPE [130]. The ability of CD36 to mediate anti-angiogenesis of TSP-1 suggests that CD36 dysfunction may cause neovascularization in addition to abnormal phagocytosis [138]. Kondo et al. used TaqMan genotyping to detect 19 single-nucleotide polymorphisms in CD36 in 109 neovascular AMD and 182 unrelated control subjects, finding that two variants in CD36, rs3173798 and rs3211883, were associated with neovascular AMD. These results suggest that CD36 may be a new candidate susceptibility gene for neovascular AMD [139]. Honda et al. used the TaqMan probe method to genotype 19 single-nucleotide polymorphisms of CD36 in 73 polypoidal choroidal vasculopathy (PCV) patients who responded to photodynamic therapy (PDT) treatment and 64 PCV patients who did not respond to treatment. The results showed that the CD36 rs3173798 variant may be associated with the visual prognosis of PCV patients with PDT [140]. As PCV is a subtype of AMD [141], this study further suggested that CD36 may provide genetic information for the development of AMD.

Studies have found that RPE cells are involved in oxidation-induced release and uptake of EVs, which can in turn act as a factor to accelerate the oxidative damage of AMD. Aged RPE cells release more RPE-cell-derived microparticles (RMPs), which can accelerate the senescence of RPE cells and interrupt the phagocytosis activity. Because blocking CD36 effectively attenuates the uptake of RMPs by RPE cells, it is speculated that CD36 on RPE accelerates the formation of AMD by participating in the uptake of RMPs [97].

**Figure 2:**
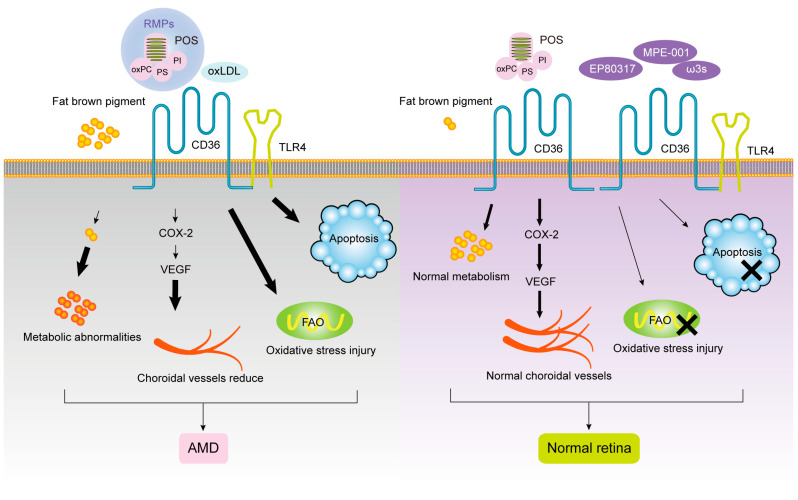
The roles of CD36 in AMD.

EP80317, a novel ligand of selective CD36, is a derivative of growth-hormone-releasing peptide-6 (GHRP-6), which lacks growth-promoting activity due to the presence of lysine 3 and is considered to have potential anti-atherogenic activity [142]. Picard et al. found significant AMD represented by increased thickness of BM in *ApoE*^−/−^ high-fat cholesterol-fed mice by electron microscopy. Later studies found that *CD36* and *ApoE* double-knockout normal-diet fed mice also showed age-related subretinal oxLDL accumulation and BM thickening, while *ApoE*^−/−^ high-fat diet mice injected with CD36 agonist EP80317 through the tail vein had the above pathological features significantly reduced and they retained some photoreceptor function. The mechanism may be that EP80317 promotes the clearance of oxidized lipids in BM and maintains photoreceptor function by increasing the expression of CD36 protein on RPE. Therefore, CD36 may be a promising therapeutic target for AMD [143]. Nitrogen impurity peptide MPE-001 (His-D-TRP-Ala-acetyl-d-Phys-NH2) is an amino derivative of GHRP-6 with high CD36 binding affinity [144]. Dorion et al. elucidated the role of MPE-001 as a CD36 ligand in the cytoprotective mechanism of RPE by applying the MPE-001 to a model of AMD oxidative stress developed from sodium iodate and RPE cell lines [11]. Kindzelskii et al. found that CD36 arrived at the POS–RPE cell interface, followed by TLR4 aggregation within 2 min, followed by metabolic and calcium signaling, suggesting that TLRs after CD36 are involved in RPE transmembrane metabolism, calcium signaling, and ROM release in RPE uptake of POS [16]. Diet plays a role as a regulator of fatty acid structure in the neurosensory retina. Promoting the intake of ω3 long-chain polyunsaturated fatty acids and decreasing the linoleic acid diet can upregulate the expression of the *CD36* gene involved in lipid transport and angiogenesis in the neurosensory retina of rats, as well as change the fatty acid profile. This is consistent with the findings of epidemiological studies related to AMD, but no effect on retinal function was observed. Therefore, the optimization of diet structure may provide a new therapeutic direction for the prevention of AMD retinal function damage through CD36. In the pathogenesis of AMD, given the important function of CD36 in POS phagocytosis by RPE, recovery/activation of CD36 expression by specific recognition of ligands may provide new ideas for the treatment of AMD. The role of CD36 ligands and signal transduction pathways in the progression of AMD is shown in Figure 2.

#### 4.1.2. CD36 and Diabetic Retinopathy (DR)

Diabetic retinopathy (DR) is a vascular abnormality including basement membrane thickening, pericyte loss, microaneurysm formation, and capillary leakage [145]. Chronic low-grade inflammation caused by abnormal expression of proinflammatory cytokines in retinal cells plays an important role in the development of DR; can adjust abnormal biological and biochemical processes of pericytes, endothelial cells, and microglia; impairs cell proliferation, endothelial cell tight junctions, and other cell function; and lead to apoptosis, ultimately causing vision loss [146]. Leukocyte adhesion to retinal vessels and proinflammatory cytokine release are two important markers of early vascular inflammation in DR [101].

COX-2 is an immediate early gene product induced by inflammatory cytokines, mitogens, and endotoxins, leading to an increase in prostaglandins during inflammation. Sennlaub et al. found that COX-2-inhibitor-induced upregulation of anti-angiogenic factor TSP-1 and CD36 receptors in endothelial cells prevented intravitreal neovascularization. Prostaglandin E2 reverses the effect of COX-2 inhibitors on TSP-1 and CD36 and aggravates the formation of intravitreal neovascularization. Moreover, wb results suggested that this effect might be independent of VEGF. Therefore, COX-2 may play an important role in ischemic proliferative retinal diseases such as diabetic retinopathy by inhibiting CD36 [147].

Modification of LDL may make it immunogenic, leading to the formation of LDL immune complexes (LDL-ICS) [148]. Elevated circulating ox-LDL-ICS levels were found to predict the risk of severe nonproliferative DR and proliferative DR in type 1 diabetes mellitus [149]. Plasma levels of malondialdehyde modified apolipoprotein B-100 antibody are positively associated with the severity of DR in type 2 diabetes mellitus, and the importance of ox-LDL-ICS is further emphasized [102]. Ox-LDL-ICS is involved in the induction of retinal oxidative stress, endoplasmic reticulum stress, and apoptosis; increases the secretion of inflammatory cytokines; and reduces the secretion of the key anti-angiogenic factor pigment epithelium-derived factor, having a toxic effect on retinal capillary pericytes. After blocking CD36, the oxidative stress level and ER-stress-mediated apoptosis of retinal pericytes were attenuated [150]. Thus, CD36 mediates the interaction of pericytes with ox-LDL-ICS, but no similar effect was found in mesangial cells [151].

Free fatty acids, especially saturated fatty acids (SFA), were found to upregulate the expression of proinflammatory cytokines [152]. Clinical studies have shown a correlation between serum levels of saturated fatty acids and the severity of DR [153], with palmitate being the most abundant SFA in humans [154]. Lu et al. found that human retinal microvascular endothelial cells (HRMVECs) expressed CD36 in vitro. It is also involved in the upregulation of IL-6 by lipopolysaccharide (LPS), palmitate, or LPS+ palmitate, which may trigger inflammatory signals such as the JNK cascade [14]. Therefore, it is speculated that HRMVECs participate in palmitate-induced signal activation and subsequent gene expression through CD36, causing DR [155].

**Figure 3:**
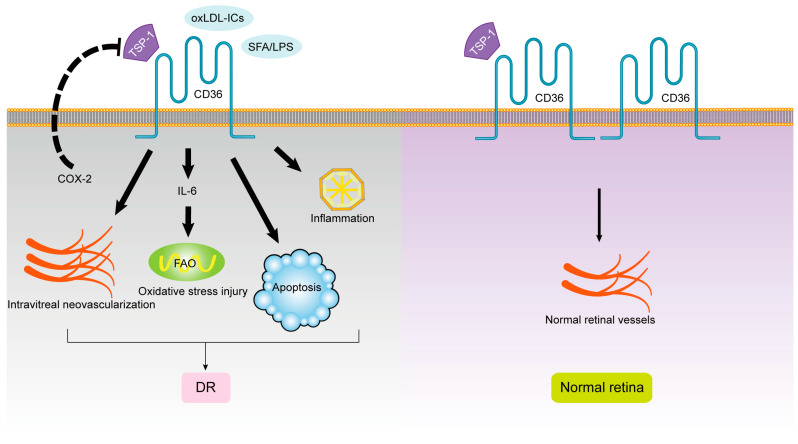
The roles of CD36 in DR.

In conclusion, lowering blood lipids, reducing oxidative stress and ox-LDL production, and antagonizing CD36 receptors may effectively prevent diabetic retinopathy. The role of CD36 ligands and signal transduction pathways in the progression of DR is shown in Figure 3.

#### 4.1.3. CD36 and Glaucoma

Glaucoma, the world’s first irreversible cause of blindness, is characterized by progressive RGC loss in retinal ganglion cells. In glaucoma and other neurodegenerative diseases, such as Alzheimer’s disease, microglia, as resident immune cells in the central nervous system, can mediate neuroinflammation; recognize and bind Aβ through membrane receptors, especially CD36; and exacerbate pathological progress [156,157].

Simons et al. found that Aβ mediates retinal microglia inflammation through CD36 activation, induced different patterns of RGC degeneration loss, and increases glial cell proliferation and activation, promoting the production of reactive oxygen species (ROS) and the secretion of proinflammatory cytokines including IL-1β and TNF-α, thereby generating intracellular signaling cascades, which may be the mechanism of retinal CD36-mediated RGC injury [104]. However, mice lacking CD36 receptors showed significantly reduced Aβ-mediated retinal RGC damage [158]. Therefore, inhibition of CD36-mediated amyloid Aβ accumulation in the retina can effectively prevent RGC loss, which may provide A new idea for the treatment of glaucoma.

#### 4.1.4. CD36 and Retinal Neovascularization

In mice born blind, vision after birth depends on the development of retinal blood vessels, and retinal hypoxia leads to neovascularization.

TSP-1 has multiple domains and interacts with many cell surface receptors in vertebrates [119]. As a stromal cell calcium-binding protein, TSP-1 regulates cytoskeletal organization, adhesion, migration, and apoptosis; limits vessel density in normal tissues; and blocks pathological angiogenesis and development, making it a naturally occurring angiogenesis inhibitor [159]. Jiménez et al. showed that as a receptor for TSP-1, CD36 is involved in the angiogenesis blocking effect of TSP-1 and activates subsequent downstream signaling pathways including p38 MAPKs, p59Fyn, and caspase-3-like proteases. Co-immunoprecipitation assay suggested that Fyn was a mediator of the negative function of TSP-1/CD36, while Src provided a positive signaling pathway promoting microvascular survival [120]. Tian et al.’s study on rhesus-macaque-derived choroid-retinal endothelial (RF/6A) cells found that VR-10 peptide (Val-Thr-Cys-Gly-Val-Ile-Thr-Arg-Ile-Arg) located at the anti-neovascularization site of TSP-1 could interact with its receptor CD36 to regulate the generation of choroidal anti-neovascularization [160].

**Figure 4:**
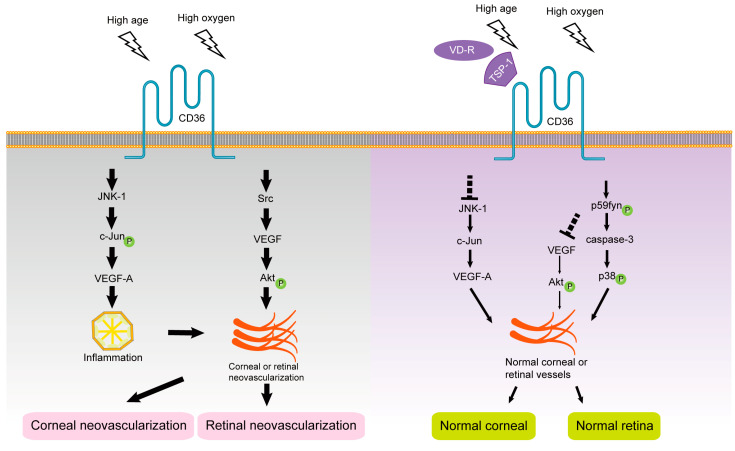
The roles of CD36 in intraocular neovascularization.

Hypoxic conditions increase the mRNA stability and expression level of VEGF, which explains the downregulation of endogenous VEGF-A expression in mouse pups under hyperoxia, leading to apoptosis of immature vascular endothelial cells that are not encapsulated by pericytes in vivo [161]. Chu et al. found that the binding of TSP-1 to CD36 promotes the binding of Src-homology-2-domain-containing protein tyrosine phosphatase-1 to CD36-vascular endothelial growth factor receptor 2 (CD36-VEGFR2) complexes in microvascular endothelial cells, attenuating VEGF signaling, dephosphorylating VEGFR2, and inhibiting angiogenesis [162]. Sun et al. used co-immunoprecipitation and other experiments to show that the presence and absence of TSP-1 recruit Fyn or Src to the CD36 membrane domain, respectively, to regulate microvascular remodeling in the developing retina by antagonizing or promoting VEGF-driven Akt signaling phosphorylation [103]. These results suggest that TSP-1 may inhibit VEGF-involved retinal neovascularization through the TSP-1/CD36/Fyn pathway, and thus CD36 may be a potential therapeutic target for retinal neovascularization. The role of CD36 ligands and signal transduction pathways in the progression of retinal neovascularization are shown in Figure 4.

#### 4.1.5. CD36 and Subretinal Inflammation

Inflammation is an important component of retinal degenerative diseases. Mononuclear phagocytic cells (MP; by monocytes, of macrophages and microglia cells) in the subretinal space lead to activation and aggregation, with proinflammatory and potential neurotoxicity, causing degeneration of RPE and photoreceptors and eventually causing retinal degenerative diseases such as diabetic retinopathy and age-related macular degeneration [163,164]. Abe et al. showed that CD36 is co-expressed on the surface of MP with a TLR2/6 heterodimer combination, participates in the clearance of various debris, maintains TLR2/6 signaling induced by diacylglycerol, and regulates TLR2-dependent macrophage-driven inflammatory response [165]. Alterations in the metabolic rate of MP, such as inhibition of glycolysis or oxidative phosphorylation, alter the activation of M1 or M2 in different inflammatory profiles, respectively [166].

**Figure 5:**
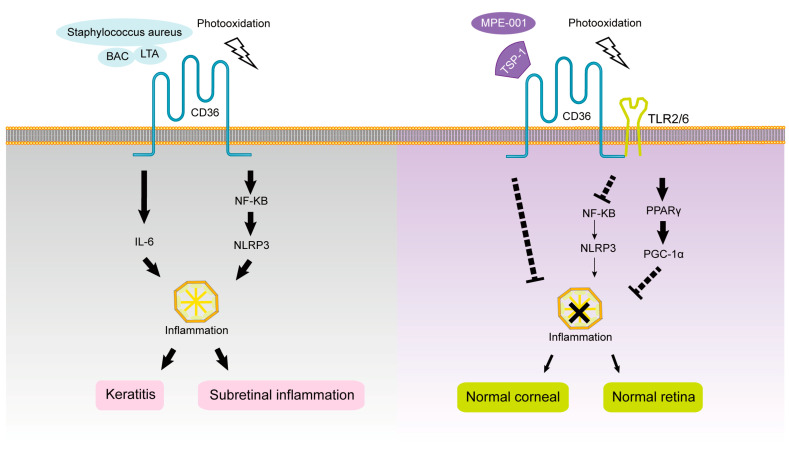
The roles of CD36 in intraocular inflammation.

Mellal et al. found that CD36-deficient mice have less subretinal MP accumulation and inflammatory cytokine infiltration and preserve the integrity and function of photoreceptor structure, which is involved in the development and treatment of degenerative retinal diseases. The CD36 selective nitrogen impurity peptide ligand MPE-001 in wild-type mice can specifically modulate CD36–TLR2 interactions to modulate the inflammatory profile and subsequent neurotoxicity of MP. MPE-001 also causes the metabolic pathway of M1-type MP to change from a glycolytic state to a state favoring oxygen consumption. Therefore, MPE-001 is expected to target the CD36 receptor and reduce chronic retinal inflammation driven by MP. MPE-001 inhibits some CD36 signaling pathways, such as NF-κB and NLRP3 inflammasome activation, and attenuates the inflammasome cascade and alters metabolic rate to increase oxygen consumption by activating other signaling pathways, such as PPARγ/PGC1α [105]. Lavalette et al. used *Cx3cr1*^−/−^ and *Cx3cr1*^−/−^*CD36*^−/−^ mice and fluorescent staining, finding that in *Cx3cr1*^−/−^ mice with light-induced subretinal inflammation, IL-6 enables mononuclear phagocytes to survive and accumulate in the subretinal under immunosuppressive conditions, causing photoreceptor cell degeneration, and this process depends on CD36. Studies suggest that CD36 may be involved in the formation of subretinal sterile inflammation [167]. The role of CD36 ligands and signal transduction pathways in the progression of intraocular inflammation are shown in Figure 5.

### 4.2. Ocular Surface Diseases and Pathological Changes

#### 4.2.1. CD36 and Corneal Neovascularization (CNV)

The angiogenic privilege of the cornea refers to the phenomenon that the cornea is normally devoid of blood vessels and actively maintains this avascular state. Although the occurrence of neovascularization is beneficial to the reconstruction of damaged tissues, good optical clarity and an unobstructed light path are very important for the maintenance of good visual acuity for some tissues or organs lacking blood vessels, such as the cornea [168]. Corneal neovascularization (CNV) extending centrally from the limbal vascular plexus blocks the passage of light, resulting in corneal opacity, irreparable damage to the structure and function of the tissue, and even the final loss of vision, and therefore blocking CNV in the cornea is very important.

The results of the A gene chip related to CNV by Ren et al. showed that serum amyloid A (SAA) is mainly produced by hepatocytes [106]. SAA and formyl peptide receptor 2 (Fpr2), which are closely related to inflammation, were upregulated, suggesting that they may be closely related to CNV, but CD36 was not involved in this pathway [107]. Mwaikambo et al. showed that the corneal epithelium constitutively expresses CD36, which acts as an endogenous antiangiogenic receptor binding to a variety of ligands including TSP-1, ox-LDL, and apoptotic cells [169]. The corneas of CD36-deficient mice showed no significant changes at 4 weeks of age but showed significant age-dependent CNV in the central cornea with corneal scar formation and corneal stromal inflammatory cell infiltration at 52 weeks of age, suggesting that CD36 can effectively inhibit inflammatory corneal neovascularization. It is speculated that this alteration is secondary to increased pathological changes such as age-dependent scar formation and chronic inflammation rather than the result of *CD36* gene knockdown alone. Because the ability of the cornea to respond to environmental or inflammatory insults declines in aged mice, the protective effect provided by CD36 becomes more important with aging [13]. This effect was associated with reduced corneal transcription of the antiangiogenic factor TSP-1 and increased mRNA levels of VEGF-A, JNK-1, and c-Jun, with activation of JNK-1 and subsequent c-Jun phosphorylation required for the angiogenic effects of VEGF-A. In addition, macrophages have been shown to provide essential VEGF for inflammatory CNV.

The role of CD36 ligands and signal transduction pathways in the progression of corneal neovascularization are shown in Figure 4.

#### 4.2.2. CD36 and Keratitis

In the cornea, epithelial cells must migrate as an intact sheet to maintain barrier function, which involves cell-to-matrix and cell-to-cell adhesion. Epithelial cells that are detached from the basement membrane undergo apoptosis, and therefore adhesion is essential for the survival of adherent cells on the basement membrane [108]. Due to the barrier function of the cornea, mice are highly resistant to infection by staphylococci, the normal bacterial flora on the ocular surface, and thus spontaneous bacterial keratitis usually does not occur [170]. However, the damage to any one of the multiple barriers that prevent bacterial adhesion and invasion can increase bacterial susceptibility [108]. CD36 is expressed in corneal and limbal epithelial cells and its primary function appears to be to maintain a physical barrier that prevents bacterial binding and is a key component of resistance to infection [171]. Yoon et al. also found that corneal fibroblasts are macrophage-derived fibroblasts, which may have the activity of macrophage adhesion and phagocytosis as well as the removal of cell debris [69]. Klocke et al. performed Lc-biotin staining of mildly deficient *CD36^−/−^* corneas and showed that the basal epithelium was detached from the basement membrane with loss of tight junctions, suggesting that cell adhesion defects may lead to the formation of mild corneal defects, signifying that CD36 is involved in maintaining the structural integrity of the corneal epithelial cells and is age related. Human corneal and conjunctival epithelial cell lines were exposed to benzalkonium chloride for 5 or 15 min as proinflammatory or proapoptotic stimuli, respectively. The established ocular surface inflammation model showed downregulation of CD36 mRNA expression, suggesting that CD36 may be involved in the inflammatory and apoptotic processes of both epithelial cells [108].

In addition to maintaining the cellular integrity of the corneal epithelium through its adhesion function and acting as a physical barrier to bacterial binding, CD36 also serves to maintain the avascular nature of the cornea and prevent CNV. In a mouse model of inflammatory CNV, CD36 inhibited CNV through indirect inhibition of macrophage-derived VEGF-A and direct inhibition of vascular growth expression [108]. Age-dependent CNV with increased expression of VEGF and inflammation has been found in *CD36*^−/−^ mice. Before neovascularization in *CD36*^−/−^ mice, a mild corneal defect occurs, characterized by loss of epithelial tight junctions, breakdown of the mucin layer, and mild macrophage infiltration in the matrix underlying the epithelial defect occurred before CNV [171]. Thus, neovascularization may occur secondary to corneal epithelial defects and subsequent association with normal microbiota in *CD36*^−/−^ mice, rather than spontaneously.

Klocke et al. found that the protective innate immune responses and the ability of macrophages to phagocytose bacteria were significantly reduced by intravenous Staphylococcus aureus in *CD36*^−/−^ mice. However, TSP1^−/−^ and TLR2^−/−^ mice did not develop spontaneous keratitis, and a new function of CD36 in maintaining the corneal epithelial barrier against infection was hypothesized to be independent of TSP-1 and TLR2, which is somewhat different from the results of Laura et al [108]. The latter study showed that exogenous TSP-1 treatment increased CD36 protein and mRNA levels in both human corneal and conjunctival epithelial cell lines, inducing inflammatory and apoptosis-related changes. However, the effect on CD36 was different. CD36 protein expression in corneal epithelial cells increased immediately after TSP-1 treatment. However, after 24 h of TSP-1 treatment, the CD36 protein expression level was not significantly different in corneal epithelial cells but increased in conjunctival epithelial cells, which may be related to the high level of secretion of TSP-1 in the basal corneal epithelium. Therefore, whether TSP-1 is involved in the development of bacterial keratitis requires further investigation [172].

The role of CD36 ligands and signal transduction pathways in the progression of keratitis are shown in Figure 5.

## 5. Conclusions

CD36 is involved in the regulation of lipid metabolism, endothelial cell function, and inflammation-related signaling pathways. Therefore, abnormal expression of CD36 in the eye can cause excessive deposition of lipofuscin and oxidized lipids under the retina, intraocular vascular abnormalities, inflammatory response, or oxidative stress damage. These characteristics make CD36 an important component in the pathogenesis of various eye diseases and a promising therapeutic target for the treatment of these eye diseases. The role of CD36 appears to be markedly different in different cell types.

On the one hand, the cell surface receptor CD36 gives RPE cells specialized phagocytic properties, and CD36 can help RPE cells bind POS containing lipid components, which makes for a good preparation for the removal of the latter and can prevent the accumulation of lipofuscin and the formation of drusen under the retina. CD36 ligands such as EP80317, MPE-001, and ω3 long-chain fatty acids can enhance the early process of intraocular lipid clearance, reduce oxidative stress damage and cell apoptosis, and delay the progression of AMD. On the other hand, in retinal microvascular endothelial cells, CD36 is involved in ocular neovascularization induced by oxidized lipid immune complexes, LPS, and hyperoxia, which is the main pathological changes leading to DR and corneal and retinal neovascularization. However, interestingly, unlike VEGF, which is involved in corneal and retinal neovascularization, COX-2 can participate in angiogenesis in the pathogenesis of DR By inhibiting the binding of TSP-1 to CD36. This process seems to occur independently of VEGF, which is consistent with the limited clinical efficacy of anti-VEGF drugs in patients with proliferative DR. Therefore, regulating the levels or activities of TSP-1 and CD36 by selective COX-2 inhibitors may be a potential therapeutic strategy for controlling intraocular neovascularization. In addition, the CD36-selective nitrogen impurity peptide ligand MPE-001, in addition to promoting CD36-mediated phagocytosis and clearance of subretinal oxidized lipids in RPE cells, protects RPE cells from oxidative stress damage, as described previously. MPE-001 can also inhibit MP cell surface CD36 receptor downstream signaling pathways that cause inflammatory response, protect the retina, and reduce MP-driven aseptic chronic inflammation and inflammation-dependent neuronal damage. This is achieved by inducing dissociation of the CD36–TLR2 oligomer complex in intraocular MP, attenuating the inflammasome cascade, and increasing the PPARγ/PGC-1α signaling pathway. This process may be related to the transition of M1-type mononuclear macrophages from a glycolytic state to a state favoring oxygen consumption. Although involved in the sterile inflammation of the retina, CD36 is a key component of resistance to keratitis caused by infection with pathogenic agents such as Staphylococcus aureus. Unlike retinal aseptic inflammation, this process does not depend on the TLR2 receptor, but whether it is related to TSP-1 is still controversial, and further research is needed. Therefore, targeted activation of the CD36 anti-inflammatory pathway may be a way to treat ocular surface and retinal inflammation.

In conclusion, the CD36 scavenger receptor is both enemy and friend in different eye diseases and cell types. Its mechanism of action is complex and plays an important role in ocular homeostasis and pathology. At present, the studies on the role of CD36 in the occurrence and development of eye diseases mainly focus on AMD, DR, etc., and it has been found that TSP-1, EP80317, MPE-001, and other proteins or derivative ligands can interact with CD36 to inhibit the pathological processes such as abnormal lipid metabolism, oxidative stress damage, or vascular abnormalities through intracellular signal transduction. It plays an important role in the prevention and treatment of the above eye diseases. However, the research on CD36 in RGC degeneration and other eye diseases and pathological changes is still in its preliminary stage, and thus it is necessary to further explore the role of CD36 and its downstream signaling pathways in the occurrence and development of a variety of eye diseases in the future. As a promising therapeutic target, the study of CD36 will help to provide early prevention and treatment for a variety of eye diseases.

## Figures and Tables

**Table 1:** The roles of CD36 in different cell types and eye diseases.

Eye Disease	Cell Type	CD36 Ligands	Pathological Damage	Refs.
AMD		POS(PtdSer)	Metabolic abnormalities, oxidative stress injury	[16,95,96]
		POS(oxPCs)	Metabolic abnormalities, oxidative stress injury	[97]
	Retinal pigment epithelium cells	POS(PS, PI)	Metabolic abnormalities	[11]
		ox-LDL, EP80317	Metabolic abnormalities	[98]
		RMPs	Metabolic abnormalities, oxidative stress injury	[99]
		MPE-001	Oxidative stress injury, autophagy	[100]
DR	Retinal microvascular endothelial cells	SFA, LPS, TSP-1	Apoptosis	[14,101]
	Periretinal cells	ox-LDL-ICS	Oxidative stress injury, apoptosis, inflammation	[102]
Retinal neovascularization	Retinal microvascular endothelial cells	TSP-1	Apoptosis	[103]
RGC degenerative injury	Retinal ganglion cells	Aβ peptide	Inflammation, oxidative stress injury	[104]
Subretinal inflammation	Mononuclear phagocytes	MPE-001	Inflammation	[105]
CNV	Corneal epithelial cell	SAA, Fpr2	Inflammation	[106,107]
Keratitis	Corneal epithelial cell	TSP-1	Inflammation	[108,109]

AMD, age-related macular degeneration; DR, diabetic retinopathy; RGC, retinal ganglion cells; CNV, corneal neovascularization; POS, photoreceptor outer segments; PtdSer, phosphatidylserines; oxPCs, oxidized phosphatidylcholines; PS, phosphatidylserine; PI, phosphatidylinositol; ox-LDL, oxidized low-density lipoprotein; RMPs, RPE-cell-derived microparticles; SFA, saturated fatty acids; LPS, lipopolysaccharide; TSP-1, thrombospondin-1; ox-LDL-ICS, oxidized low-density lipoprotein immune complexes; SAA, serum amyloid A; Fpr2, formyl peptide receptor 2.

## References

[1] Goldstein J.L., Ho Y.K., Basu S.K., Brown M.S. (1979). Binding site on macrophages that mediates uptake and degradation of acetylated low density lipoprotein, producing massive cholesterol deposition. Proc. Natl. Acad. Sci. USA.

[2] Brown M.S., Goldstein J.L. (1983). Lipoprotein metabolism in the macrophage: Implications for cholesterol deposition in atherosclerosis. Annu. Rev. Biochem..

[3] PrabhuDas M.R., Baldwin C.L., Bollyky P.L., Bowdish D.M.E., Drickamer K., Febbraio M., Herz J., Kobzik L., Krieger M., Loike J. (2017). A Consensus Definitive Classification of Scavenger Receptors and Their Roles in Health and Disease. J. Immunol..

[4] Prabhudas M., Bowdish D., Drickamer K., Febbraio M., Herz J., Kobzik L., Krieger M., Loike J., Means T.K., Moestrup S.K. (2014). Standardizing scavenger receptor nomenclature. J. Immunol..

[5] Hsieh F.L., Turner L., Bolla J.R., Robinson C.V., Lavstsen T., Higgins M.K. (2016). The structural basis for CD36 binding by the malaria parasite. Nat. Commun..

[6] Yang X., Okamura D.M., Lu X., Chen Y., Moorhead J., Varghese Z., Ruan X.Z. (2017). CD36 in chronic kidney disease: Novel insights and therapeutic opportunities. Nat. Rev. Nephrol..

[7] Atzeni F., Rodríguez-Carrio J., Popa C.D., Nurmohamed M.T., Szűcs G., Szekanecz Z. (2021). Cardiovascular effects of approved drugs for rheumatoid arthritis. Nat. Rev. Rheumatol..

[8] Li J., Yu C., Wang R., Xu J., Chi Y., Qin J., Liu Q. (2017). The ω-carboxyl group of 7-ketocholesteryl-9-carboxynonanoate mediates the binding of oxLDL to CD36 receptor and enhances caveolin-1 expression in macrophages. Int. J. Biochem. Cell Biol..

[9] Moon J.S., Karunakaran U., Suma E., Chung S.M., Won K.C. (2020). The Role of CD36 in Type 2 Diabetes Mellitus: β-Cell Dysfunction and Beyond. Diabetes Metab. J..

[10] Rada P., González-Rodríguez Á., García-Monzón C., Valverde Á.M. (2020). Understanding lipotoxicity in NAFLD pathogenesis: Is CD36 a key driver?. Cell Death Dis..

[11] Dorion M.F., Mulumba M., Kasai S., Itoh K., Lubell W.D., Ong H. (2021). The CD36 Ligand-Promoted Autophagy Protects Retinal Pigment Epithelial Cells from Oxidative Stress. Oxidative Med. Cell. Longev..

[12] Roggia M.F., Ueta T. (2015). αvβ5 Integrin/FAK/PGC-1α Pathway Confers Protective Effects on Retinal Pigment Epithelium. PLoS ONE.

[13] Mwaikambo B.R., Sennlaub F., Ong H., Chemtob S., Hardy P. (2008). Genetic ablation of CD36 induces age-related corneal neovascularization. Cornea.

[14] Lu Z., Li Y., Ru J.H., Lopes-Virella M.F., Lyons T.J., Huang Y. (2019). Interaction of palmitate and LPS regulates cytokine expression and apoptosis through sphingolipids in human retinal microvascular endothelial cells. Exp. Eye Res..

[15] Stewart C.R., Stuart L.M., Wilkinson K., van Gils J.M., Deng J., Halle A., Rayner K.J., Boyer L., Zhong R., Frazier W.A. (2010). CD36 ligands promote sterile inflammation through assembly of a Toll-like receptor 4 and 6 heterodimer. Nat. Immunol..

[16] Kindzelskii A.L., Elner V.M., Elner S.G., Yang D., Hughes B.A., Petty H.R. (2004). Toll-like receptor 4 (TLR4) of retinal pigment epithelial cells participates in transmembrane signaling in response to photoreceptor outer segments. J. Gen. Physiol..

[17] Mast N., Bederman I.R., Pikuleva I.A. (2018). Retinal Cholesterol Content Is Reduced in Simvastatin-Treated Mice Due to Inhibited Local Biosynthesis Albeit Increased Uptake of Serum Cholesterol. Drug Metab. Dispos. Biol. Fate Chem..

[18] Collot-Teixeira S., Martin J., McDermott-Roe C., Poston R., McGregor J.L. (2007). CD36 and macrophages in atherosclerosis. Cardiovasc. Res..

[19] Armesilla A.L., Vega M.A. (1994). Structural organization of the gene for human CD36 glycoprotein. J. Biol. Chem..

[20] Qiao L., Zou C., Shao P., Schaack J., Johnson P.F., Shao J. (2008). Transcriptional regulation of fatty acid translocase/CD36 expression by CCAAT/enhancer-binding protein alpha. J. Biol. Chem..

[21] Wang J., Qin X., Sun H., He M., Lv Q., Gao C., He X., Liao H. (2021). Nogo receptor impairs the clearance of fibril amyloid-β by microglia and accelerates Alzheimer’s-like disease progression. Aging Cell.

[22] Xu J., Yu T., Pietronigro E.C., Yuan J., Arioli J., Pei Y., Luo X., Ye J., Constantin G., Mao C. (2020). Peli1 impairs microglial Aβ phagocytosis through promoting C/EBPβ degradation. PLoS Biol..

[23] Kim S.M., Mun B.R., Lee S.J., Joh Y., Lee H.Y., Ji K.Y., Choi H.R., Lee E.H., Kim E.M., Jang J.H. (2017). TREM2 promotes Aβ phagocytosis by upregulating C/EBPα-dependent CD36 expression in microglia. Sci. Rep..

[24] Dai Y., Su W., Ding Z., Wang X., Mercanti F., Chen M., Raina S., Mehta J.L. (2013). Regulation of MSR-1 and CD36 in macrophages by LOX-1 mediated through PPAR-γ. Biochem. Biophys. Res. Commun..

[25] Goto K., Iso T., Hanaoka H., Yamaguchi A., Suga T., Hattori A., Irie Y., Shinagawa Y., Matsui H., Syamsunarno M.R. (2013). Peroxisome proliferator-activated receptor-γ in capillary endothelia promotes fatty acid uptake by heart during long-term fasting. J. Am. Heart Assoc..

[26] Feng L., Gu C., Li Y., Huang J. (2017). High Glucose Promotes CD36 Expression by Upregulating Peroxisome Proliferator-Activated Receptor γ Levels to Exacerbate Lipid Deposition in Renal Tubular Cells. BioMed Res. Int..

[27] Luiken J.J., Chanda D., Nabben M., Neumann D., Glatz J.F. (2016). Post-translational modifications of CD36 (SR-B2): Implications for regulation of myocellular fatty acid uptake. Biochim. Et Biophys. Acta.

[28] Abumrad N.A., el-Maghrabi M.R., Amri E.Z., Lopez E., Grimaldi P.A. (1993). Cloning of a rat adipocyte membrane protein implicated in binding or transport of long-chain fatty acids that is induced during preadipocyte differentiation. Homology with human CD36. J. Biol. Chem..

[29] Cao D., Luo J., Chen D., Xu H., Shi H., Jing X., Zang W. (2016). CD36 regulates lipopolysaccharide-induced signaling pathways and mediates the internalization of Escherichia coli in cooperation with TLR4 in goat mammary gland epithelial cells. Sci Rep..

[30] Xing Q., Feng Y., Sun H., Yang S., Sun T., Guo X., Ji F., Wu B., Zhou D. (2021). Scavenger receptor MARCO contributes to macrophage phagocytosis and clearance of tumor cells. Exp. Cell Res..

[31] Thorne R.F., Ralston K.J., de Bock C.E., Mhaidat N.M., Zhang X.D., Boyd A.W., Burns G.F. (2010). Palmitoylation of CD36/FAT regulates the rate of its post-transcriptional processing in the endoplasmic reticulum. Biochim. Et Biophys. Acta.

[32] Hao J.W., Wang J., Guo H., Zhao Y.Y., Sun H.H., Li Y.F., Lai X.Y., Zhao N., Wang X., Xie C. (2020). CD36 facilitates fatty acid uptake by dynamic palmitoylation-regulated endocytosis. Nat. Commun..

[33] Zeng S., Wu F., Chen M., Li Y., You M., Zhang Y., Yang P., Wei L., Ruan X.Z., Zhao L. (2022). Inhibition of Fatty Acid Translocase (FAT/CD36) Palmitoylation Enhances Hepatic Fatty Acid β-Oxidation by Increasing Its Localization to Mitochondria and Interaction with Long-Chain Acyl-CoA Synthetase 1. Antioxid. Redox Signal..

[34] Aicart-Ramos C., Valero R.A., Rodriguez-Crespo I. (2011). Protein palmitoylation and subcellular trafficking. Biochim. Et Biophys. Acta.

[35] Meiler S., Baumer Y., Huang Z., Hoffmann F.W., Fredericks G.J., Rose A.H., Norton R.L., Hoffmann P.R., Boisvert W.A. (2013). Selenoprotein K is required for palmitoylation of CD36 in macrophages: Implications in foam cell formation and atherogenesis. J. Leukoc. Biol..

[36] van Oort M.M., Drost R., Janβen L., Van Doorn J.M., Kerver J., Van der Horst D.J., Luiken J.J., Rodenburg K.C. (2014). Each of the four intracellular cysteines of CD36 is essential for insulin- or AMP-activated protein kinase-induced CD36 translocation. Arch. Physiol. Biochem..

[37] Zhang Y., Dong D., Xu X., He H., Zhu Y., Lei T., Ou H. (2022). Oxidized high-density lipoprotein promotes CD36 palmitoylation and increases lipid uptake in macrophages. J. Biol. Chem..

[38] Popovic D., Vucic D., Dikic I. (2014). Ubiquitination in disease pathogenesis and treatment. Nat. Med..

[39] Smith J., Su X., El-Maghrabi R., Stahl P.D., Abumrad N.A. (2008). Opposite regulation of CD36 ubiquitination by fatty acids and insulin: Effects on fatty acid uptake. J. Biol. Chem..

[40] Wu W., Wang S., Liu Q., Shan T., Wang X., Feng J., Wang Y. (2020). AMPK facilitates intestinal long-chain fatty acid uptake by manipulating CD36 expression and translocation. FASEB J..

[41] Kim K.Y., Stevens M.V., Akter M.H., Rusk S.E., Huang R.J., Cohen A., Noguchi A., Springer D., Bocharov A.V., Eggerman T.L. (2011). Parkin is a lipid-responsive regulator of fat uptake in mice and mutant human cells. J. Clin. Investig..

[42] Xia X., Hu T., He J., Xu Q., Yu C., Liu X., Shao Z., Liao Y., Huang H., Liu N. (2020). USP10 deletion inhibits macrophage-derived foam cell formation and cellular-oxidized low density lipoprotein uptake by promoting the degradation of CD36. Aging.

[43] Zhang F., Xia X., Chai R., Xu R., Xu Q., Liu M., Chen X., Liu B., Liu S., Liu N. (2020). Inhibition of USP14 suppresses the formation of foam cell by promoting CD36 degradation. J. Cell. Mol. Med..

[44] Xia X., Xu Q., Liu M., Chen X., Liu X., He J., Hu T., Yu C., Huang H., Liu S. (2020). Deubiquitination of CD36 by UCHL1 promotes foam cell formation. Cell Death Dis..

[45] Moremen K.W., Tiemeyer M., Nairn A.V. (2012). Vertebrate protein glycosylation: Diversity, synthesis and function. Nat. Rev. Mol. Cell Biol..

[46] Hoosdally S.J., Andress E.J., Wooding C., Martin C.A., Linton K.J. (2009). The Human Scavenger Receptor CD36: Glycosylation status and its role in trafficking and function. J. Biol. Chem..

[47] Lauzier B., Merlen C., Vaillant F., McDuff J., Bouchard B., Beguin P.C., Dolinsky V.W., Foisy S., Villeneuve L.R., Labarthe F. (2011). Post-translational modifications, a key process in CD36 function: Lessons from the spontaneously hypertensive rat heart. J. Mol. Cell. Cardiol..

[48] Jiang M., Wu N., Xu B., Chu Y., Li X., Su S., Chen D., Li W., Shi Y., Gao X. (2019). Fatty acid-induced CD36 expression via O-GlcNAcylation drives gastric cancer metastasis. Theranostics.

[49] Viñals M., Xu S., Vasile E., Krieger M. (2003). Identification of the N-linked glycosylation sites on the high density lipoprotein (HDL) receptor SR-BI and assessment of their effects on HDL binding and selective lipid uptake. J. Biol. Chem..

[50] Laczy B., Fülöp N., Onay-Besikci A., Des Rosiers C., Chatham J.C. (2011). Acute regulation of cardiac metabolism by the hexosamine biosynthesis pathway and protein O-GlcNAcylation. PLoS ONE.

[51] Yamamoto Y., Hiasa Y., Murakami H., Ikeda Y., Yamanishi H., Abe M., Matsuura B., Onji M. (2012). Rapid alternative absorption of dietary long-chain fatty acids with upregulation of intestinal glycosylated CD36 in liver cirrhosis. Am. J. Clin. Nutr..

[52] Chu L.Y., Silverstein R.L. (2012). CD36 ectodomain phosphorylation blocks thrombospondin-1 binding: Structure-function relationships and regulation by protein kinase C. Arterioscler. Thromb. Vasc. Biol..

[53] Hatmi M., Gavaret J.M., Elalamy I., Vargaftig B.B., Jacquemin C. (1996). Evidence for cAMP-dependent platelet ectoprotein kinase activity that phosphorylates platelet glycoprotein IV (CD36). J. Biol. Chem..

[54] Lynes M., Narisawa S., Millán J.L., Widmaier E.P. (2011). Interactions between CD36 and global intestinal alkaline phosphatase in mouse small intestine and effects of high-fat diet. Am. J. Physiol. Regul. Integr. Comp. Physiol..

[55] Asch A.S., Liu I., Briccetti F.M., Barnwell J.W., Kwakye-Berko F., Dokun A., Goldberger J., Pernambuco M. (1993). Analysis of CD36 binding domains: Ligand specificity controlled by dephosphorylation of an ectodomain. Science.

[56] Guthmann F., Maehl P., Preiss J., Kolleck I., Rüstow B. (2002). Ectoprotein kinase-mediated phosphorylation of FAT/CD36 regulates palmitate uptake by human platelets. Cell. Mol. Life Sci..

[57] Pfleger J., Gross P., Johnson J., Carter R.L., Gao E., Tilley D.G., Houser S.R., Koch W.J. (2018). G protein-coupled receptor kinase 2 contributes to impaired fatty acid metabolism in the failing heart. J. Mol. Cell. Cardiol..

[58] Lundby A., Lage K., Weinert B.T., Bekker-Jensen D.B., Secher A., Skovgaard T., Kelstrup C.D., Dmytriyev A., Choudhary C., Lundby C. (2012). Proteomic analysis of lysine acetylation sites in rat tissues reveals organ specificity and subcellular patterns. Cell Rep..

[59] Kuda O., Pietka T.A., Demianova Z., Kudova E., Cvacka J., Kopecky J., Abumrad N.A. (2013). Sulfo-N-succinimidyl oleate (SSO) inhibits fatty acid uptake and signaling for intracellular calcium via binding CD36 lysine 164: SSO also inhibits oxidized low density lipoprotein uptake by macrophages. J. Biol. Chem..

[60] Khan S., Kowluru A. (2018). CD36 mediates lipid accumulation in pancreatic beta cells under the duress of glucolipotoxic conditions: Novel roles of lysine deacetylases. Biochem. Biophys. Res. Commun..

[61] Du X., Jiang S., Zeng X., Zhang J., Pan K., Zhou J., Xie Y., Kan H., Song W., Sun Q. (2018). Air pollution is associated with the development of atherosclerosis via the cooperation of CD36 and NLRP3 inflammasome in ApoE(-/-) mice. Toxicol Lett.

[62] Karunakaran U., Elumalai S., Moon J.S., Won K.C. (2021). CD36 Signal Transduction in Metabolic Diseases: Novel Insights and Therapeutic Targeting. Cells.

[63] Bonen A., Luiken J.J., Arumugam Y., Glatz J.F., Tandon N.N. (2000). Acute regulation of fatty acid uptake involves the cellular redistribution of fatty acid translocase. J. Biol. Chem..

[64] Chabowski A., Coort S.L., Calles-Escandon J., Tandon N.N., Glatz J.F., Luiken J.J., Bonen A. (2004). Insulin stimulates fatty acid transport by regulating expression of FAT/CD36 but not FABPpm. Am. J. Physiol. Endocrinol. Metab..

[65] Jeppesen J., Albers P.H., Rose A.J., Birk J.B., Schjerling P., Dzamko N., Steinberg G.R., Kiens B. (2011). Contraction-induced skeletal muscle FAT/CD36 trafficking and FA uptake is AMPK independent. J. Lipid Res..

[66] Houssier M., Raoul W., Lavalette S., Keller N., Guillonneau X., Baragatti B., Jonet L., Jeanny J.C., Behar-Cohen F., Coceani F. (2008). CD36 deficiency leads to choroidal involution via COX2 down-regulation in rodents. PLoS Med..

[67] Tserentsoodol N., Gordiyenko N.V., Pascual I., Lee J.W., Fliesler S.J., Rodriguez I.R. (2006). Intraretinal lipid transport is dependent on high density lipoprotein-like particles and class B scavenger receptors. Mol. Vis..

[68] Kang H.M., Ahn S.H., Choi P., Ko Y.A., Han S.H., Chinga F., Park A.S., Tao J., Sharma K., Pullman J. (2015). Defective fatty acid oxidation in renal tubular epithelial cells has a key role in kidney fibrosis development. Nat. Med..

[69] Yoon H., Choi S.I., Kim E.K. (2020). Uptake of cell debris and enhanced expression of inflammatory factors in response to dead cells in corneal fibroblast cells. Exp. Eye Res..

[70] Soriano-Romaní L., Contreras-Ruiz L., García-Posadas L., López-García A., Masli S., Diebold Y. (2015). Inflammatory Cytokine-Mediated Regulation of Thrombospondin-1 and CD36 in Conjunctival Cells. J. Ocul. Pharmacol. Ther. Off. J. Assoc. Ocul. Pharmacol. Ther..

[71] Tanaka T., Nakata T., Oka T., Ogawa T., Okamoto F., Kusaka Y., Sohmiya K., Shimamoto K., Itakura K. (2001). Defect in human myocardial long-chain fatty acid uptake is caused by FAT/CD36 mutations. J. Lipid Res..

[72] Wintergerst E.S., Jelk J., Rahner C., Asmis R. (2000). Apoptosis induced by oxidized low density lipoprotein in human monocyte-derived macrophages involves CD36 and activation of caspase-3. Eur J. Biochem.

[73] Liu W., Yin Y., Zhou Z., He M., Dai Y. (2014). OxLDL-induced IL-1 beta secretion promoting foam cells formation was mainly via CD36 mediated ROS production leading to NLRP3 inflammasome activation. Inflamm. Res..

[74] Seimon T.A., Nadolski M.J., Liao X., Magallon J., Nguyen M., Feric N.T., Koschinsky M.L., Harkewicz R., Witztum J.L., Tsimikas S. (2010). Atherogenic lipids and lipoproteins trigger CD36-TLR2-dependent apoptosis in macrophages undergoing endoplasmic reticulum stress. Cell Metab.

[75] Iwao Y., Nakajou K., Nagai R., Kitamura K., Anraku M., Maruyama T., Otagiri M. (2008). CD36 is one of important receptors promoting renal tubular injury by advanced oxidation protein products. Am. J. Physiol Ren. Physiol.

[76] Zhu W., Li W., Silverstein R.L. (2012). Advanced glycation end products induce a prothrombotic phenotype in mice via interaction with platelet CD36. Blood.

[77] Kuijpers M.J., de Witt S., Nergiz-Unal R., van Kruchten R., Korporaal S.J., Verhamme P., Febbraio M., Tjwa M., Voshol P.J., Hoylaerts M.F. (2014). Supporting roles of platelet thrombospondin-1 and CD36 in thrombus formation on collagen. Arterioscler. Thromb. Vasc. Biol..

[78] Simantov R., Febbraio M., Silverstein R.L. (2005). The antiangiogenic effect of thrombospondin-2 is mediated by CD36 and modulated by histidine-rich glycoprotein. Matrix Biol..

[79] Kerkhoff C., Sorg C., Tandon N.N., Nacken W. (2001). Interaction of S100A8/S100A9-arachidonic acid complexes with the scavenger receptor CD36 may facilitate fatty acid uptake by endothelial cells. Biochemistry.

[80] Tondera C., Laube M., Pietzsch J. (2017). Insights into binding of S100 proteins to scavenger receptors: Class B scavenger receptor CD36 binds S100A12 with high affinity. Amino Acids.

[81] Park L., Zhou J., Zhou P., Pistick R., El Jamal S., Younkin L., Pierce J., Arreguin A., Anrather J., Younkin S.G. (2013). Innate immunity receptor CD36 promotes cerebral amyloid angiopathy. Proc. Natl. Acad. Sci. USA.

[82] Rodrigue-Way A., Caron V., Bilodeau S., Keil S., Hassan M., Lévy E., Mitchell G.A., Tremblay A. (2014). Scavenger receptor CD36 mediates inhibition of cholesterol synthesis via activation of the PPARγ/PGC-1α pathway and Insig1/2 expression in hepatocytes. FASEB J. Off. Publ. Fed. Am. Soc. Exp. Biol..

[83] Marleau S., Harb D., Bujold K., Avallone R., Iken K., Wang Y., Demers A., Sirois M.G., Febbraio M., Silverstein R.L. (2005). EP 80317, a ligand of the CD36 scavenger receptor, protects apolipoprotein E-deficient mice from developing atherosclerotic lesions. FASEB J..

[84] Xu S., Jay A., Brunaldi K., Huang N., Hamilton J.A. (2013). CD36 enhances fatty acid uptake by increasing the rate of intracellular esterification but not transport across the plasma membrane. Biochemistry.

[85] Park Y.M. (2014). CD36, a scavenger receptor implicated in atherosclerosis. Exp. Mol. Med..

[86] Savill J., Hogg N., Ren Y., Haslett C. (1992). Thrombospondin cooperates with CD36 and the vitronectin receptor in macrophage recognition of neutrophils undergoing apoptosis. J. Clin. Investig..

[87] Navazo M.D., Daviet L., Savill J., Ren Y., Leung L.L., McGregor J.L. (1996). Identification of a domain (155-183) on CD36 implicated in the phagocytosis of apoptotic neutrophils. J. Biol. Chem..

[88] Dawson D.W., Pearce S.F., Zhong R., Silverstein R.L., Frazier W.A., Bouck N.P. (1997). CD36 mediates the In vitro inhibitory effects of thrombospondin-1 on endothelial cells. J. Cell Biol..

[89] Baruch D.I., Ma X.C., Pasloske B., Howard R.J., Miller L.H. (1999). CD36 peptides that block cytoadherence define the CD36 binding region for Plasmodium falciparum-infected erythrocytes. Blood.

[90] Puente Navazo M.D., Daviet L., Ninio E., McGregor J.L. (1996). Identification on human CD36 of a domain (155-183) implicated in binding oxidized low-density lipoproteins (Ox-LDL). Arterioscler. Thromb. Vasc. Biol..

[91] Pepino M.Y., Kuda O., Samovski D., Abumrad N.A. (2014). Structure-function of CD36 and importance of fatty acid signal transduction in fat metabolism. Annu. Rev. Nutr..

[92] Ohgami N., Nagai R., Ikemoto M., Arai H., Kuniyasu A., Horiuchi S., Nakayama H. (2001). CD36, a member of class B scavenger receptor family, is a receptor for advanced glycation end products. Ann. N. Y. Acad. Sci..

[93] Demers A., McNicoll N., Febbraio M., Servant M., Marleau S., Silverstein R., Ong H. (2004). Identification of the growth hormone-releasing peptide binding site in CD36: A photoaffinity cross-linking study. Biochem J..

[94] Pearce S.F., Roy P., Nicholson A.C., Hajjar D.P., Febbraio M., Silverstein R.L. (1998). Recombinant glutathione S-transferase/CD36 fusion proteins define an oxidized low density lipoprotein-binding domain. J. Biol. Chem..

[95] Rieu Q., Bougoüin A., Zagar Y., Chatagnon J., Hamieh A., Enderlin J., Huby T., Nandrot E.F. (2022). Pleiotropic Roles of Scavenger Receptors in Circadian Retinal Phagocytosis: A New Function for Lysosomal SR-B2/LIMP-2 at the RPE Cell Surface. Int. J. Mol. Sci..

[96] Nandrot E.F., Kim Y., Brodie S.E., Huang X., Sheppard D., Finnemann S.C. (2004). Loss of synchronized retinal phagocytosis and age-related blindness in mice lacking alphavbeta5 integrin. J. Exp. Med..

[97] Yang C., Shani S., Tahiri H., Ortiz C., Gu M., Lavoie J.C., Croteau S., Hardy P. (2020). Extracellular microparticles exacerbate oxidative damage to retinal pigment epithelial cells. Exp. Cell Res..

[98] Vollrath D., Feng W., Duncan J.L., Yasumura D., D’Cruz P.M., Chappelow A., Matthes M.T., Kay M.A., LaVail M.M. (2001). Correction of the retinal dystrophy phenotype of the RCS rat by viral gene transfer of Mertk. Proc. Natl. Acad. Sci. USA.

[99] Wu Z., Wang S., Sorenson C.M., Sheibani N. (2006). Attenuation of retinal vascular development and neovascularization in transgenic mice over-expressing thrombospondin-1 in the lens. Dev. Dyn. Off. Publ. Am. Assoc. Anat..

[100] Rocha D.M., Caldas A.P., Oliveira L.L., Bressan J., Hermsdorff H.H. (2016). Saturated fatty acids trigger TLR4-mediated inflammatory response. Atherosclerosis.

[101] Adamiec-Mroczek J., Oficjalska-Młyńczak J., Misiuk-Hojło M. (2010). Roles of endothelin-1 and selected proinflammatory cytokines in the pathogenesis of proliferative diabetic retinopathy: Analysis of vitreous samples. Cytokine.

[102] Fredrikson G.N., Anand D.V., Hopkins D., Corder R., Alm R., Bengtsson E., Shah P.K., Lahiri A., Nilsson J. (2009). Associations between autoantibodies against apolipoprotein B-100 peptides and vascular complications in patients with type 2 diabetes. Diabetologia.

[103] Sun J., Hopkins B.D., Tsujikawa K., Perruzzi C., Adini I., Swerlick R., Bornstein P., Lawler J., Benjamin L.E. (2009). Thrombospondin-1 modulates VEGF-A-mediated Akt signaling and capillary survival in the developing retina. Am. J. Physiol. Heart Circ. Physiol..

[104] Howlett D.R., Bate S.T., Collier S., Lawman A., Chapman T., Ashmeade T., Marshall I., Anderson P.J., Philpott K.L., Richardson J.C. (2011). Characterisation of amyloid-induced inflammatory responses in the rat retina. Exp. Brain Res..

[105] Mellal K., Omri S., Mulumba M., Tahiri H., Fortin C., Dorion M.F., Pham H., Garcia Ramos Y., Zhang J., Pundir S. (2019). Immunometabolic modulation of retinal inflammation by CD36 ligand. Sci. Rep..

[106] Ren S.W., Qi X., Jia C.K., Wang Y.Q. (2014). Serum amyloid A and pairing formyl peptide receptor 2 are expressed in corneas and involved in inflammation-mediated neovascularization. Int. J. Ophthalmol..

[107] Jia C., Zhu W., Ren S., Xi H., Li S., Wang Y. (2011). Comparison of genome-wide gene expression in suture- and alkali burn-induced murine corneal neovascularization. Mol. Vis..

[108] Klocke J., Barcia R.N., Heimer S., Cario E., Zieske J., Gilmore M.S., Ksander B.R., Gregory M.S. (2011). Spontaneous bacterial keratitis in CD36 knockout mice. Investig. Ophthalmol. Vis. Sci..

[109] Zhao L., Varghese Z., Moorhead J.F., Chen Y., Ruan X.Z. (2018). CD36 and lipid metabolism in the evolution of atherosclerosis. Br. Med. Bull..

[110] Elsøe S., Ahnström J., Christoffersen C., Hoofnagle A.N., Plomgaard P., Heinecke J.W., Binder C.J., Björkbacka H., Dahlbäck B., Nielsen L.B. (2012). Apolipoprotein M binds oxidized phospholipids and increases the antioxidant effect of HDL. Atherosclerosis.

[111] Dhungana H., Huuskonen M.T., Jaronen M., Lemarchant S., Ali H., Keksa-Goldsteine V., Goldsteins G., Kanninen K.M., Koistinaho J., Malm T. (2017). Sulfosuccinimidyl oleate sodium is neuroprotective and alleviates stroke-induced neuroinflammation. J. Neuroinflammation.

[112] Noushmehr H., D’Amico E., Farilla L., Hui H., Wawrowsky K.A., Mlynarski W., Doria A., Abumrad N.A., Perfetti R. (2005). Fatty acid translocase (FAT/CD36) is localized on insulin-containing granules in human pancreatic beta-cells and mediates fatty acid effects on insulin secretion. Diabetes.

[113] Lee J., Jung E., Lee J., Huh S., Kim Y.S., Kim Y.W., Kim Y.S., Park D. (2010). Anti-adipogenesis by 6-thioinosine is mediated by downregulation of PPAR gamma through JNK-dependent upregulation of iNOS. Cell. Mol. Life Sci..

[114] Daquinag A.C., Gao Z., Fussell C., Immaraj L., Pasqualini R., Arap W., Akimzhanov A.M., Febbraio M., Kolonin M.G. (2021). Fatty acid mobilization from adipose tissue is mediated by CD36 posttranslational modifications and intracellular trafficking. JCI Insight.

[115] Son N.H., Basu D., Samovski D., Pietka T.A., Peche V.S., Willecke F., Fang X., Yu S.Q., Scerbo D., Chang H.R. (2018). Endothelial cell CD36 optimizes tissue fatty acid uptake. J. Clin. Investig..

[116] Albert M.L., Pearce S.F., Francisco L.M., Sauter B., Roy P., Silverstein R.L., Bhardwaj N. (1998). Immature dendritic cells phagocytose apoptotic cells via alphavbeta5 and CD36, and cross-present antigens to cytotoxic T lymphocytes. J. Exp. Med..

[117] Rhoads J.P., Lukens J.R., Wilhelm A.J., Moore J.L., Mendez-Fernandez Y., Kanneganti T.D., Major A.S. (2017). Oxidized Low-Density Lipoprotein Immune Complex Priming of the Nlrp3 Inflammasome Involves TLR and FcγR Cooperation and Is Dependent on CARD9. J. Immunol..

[118] Ekici M., Kisa U., Arikan Durmaz S., Ugur E., Nergiz-Unal R. (2018). Fatty acid transport receptor soluble CD36 and dietary fatty acid pattern in type 2 diabetic patients: A comparative study. Br. J. Nutr..

[119] Lawler J. (2000). The functions of thrombospondin-1 and-2. Curr. Opin. Cell Biol..

[120] Jiménez B., Volpert O.V., Crawford S.E., Febbraio M., Silverstein R.L., Bouck N. (2000). Signals leading to apoptosis-dependent inhibition of neovascularization by thrombospondin-1. Nat. Med..

[121] Blasiak J. (2020). Senescence in the pathogenesis of age-related macular degeneration. Cell. Mol. Life Sci..

[122] Arandjelovic S., Ravichandran K.S. (2015). Phagocytosis of apoptotic cells in homeostasis. Nat. Immunol..

[123] Segawa K., Nagata S. (2015). An Apoptotic ’Eat Me’ Signal: Phosphatidylserine Exposure. Trends Cell Biol..

[124] Penberthy K.K., Lysiak J.J., Ravichandran K.S. (2018). Rethinking Phagocytes: Clues from the Retina and Testes. Trends Cell Biol..

[125] Kaarniranta K., Sinha D., Blasiak J., Kauppinen A., Veréb Z., Salminen A., Boulton M.E., Petrovski G. (2013). Autophagy and heterophagy dysregulation leads to retinal pigment epithelium dysfunction and development of age-related macular degeneration. Autophagy.

[126] Ryeom S.W., Silverstein R.L., Scotto A., Sparrow J.R. (1996). Binding of anionic phospholipids to retinal pigment epithelium may be mediated by the scavenger receptor CD36. J. Biol. Chem..

[127] Ren Y., Silverstein R.L., Allen J., Savill J. (1995). CD36 gene transfer confers capacity for phagocytosis of cells undergoing apoptosis. J. Exp. Med..

[128] Lin H., Clegg D.O. (1998). Integrin alphavbeta5 participates in the binding of photoreceptor rod outer segments during phagocytosis by cultured human retinal pigment epithelium. Investig. Ophthalmol. Vis. Sci..

[129] Duncan K.G., Bailey K.R., Kane J.P., Schwartz D.M. (2002). Human retinal pigment epithelial cells express scavenger receptors BI and BII. Biochem. Biophys. Res. Commun..

[130] Westenskow P.D., Moreno S.K., Krohne T.U., Kurihara T., Zhu S., Zhang Z.N., Zhao T., Xu Y., Ding S., Friedlander M. (2012). Using flow cytometry to compare the dynamics of photoreceptor outer segment phagocytosis in iPS-derived RPE cells. Investig. Ophthalmol. Vis. Sci..

[131] Finnemann S.C., Silverstein R.L. (2001). Differential roles of CD36 and alphavbeta5 integrin in photoreceptor phagocytosis by the retinal pigment epithelium. J. Exp. Med..

[132] Chang Y., Finnemann S.C. (2007). Tetraspanin CD81 is required for the alpha v beta5-integrin-dependent particle-binding step of RPE phagocytosis. J. Cell Sci..

[133] Sun M., Finnemann S.C., Febbraio M., Shan L., Annangudi S.P., Podrez E.A., Hoppe G., Darrow R., Organisciak D.T., Salomon R.G. (2006). Light-induced oxidation of photoreceptor outer segment phospholipids generates ligands for CD36-mediated phagocytosis by retinal pigment epithelium: A potential mechanism for modulating outer segment phagocytosis under oxidant stress conditions. J. Biol. Chem..

[134] Podrez E.A., Poliakov E., Shen Z., Zhang R., Deng Y., Sun M., Finton P.J., Shan L., Febbraio M., Hajjar D.P. (2002). A novel family of atherogenic oxidized phospholipids promotes macrophage foam cell formation via the scavenger receptor CD36 and is enriched in atherosclerotic lesions. J. Biol. Chem..

[135] Gordiyenko N., Campos M., Lee J.W., Fariss R.N., Sztein J., Rodriguez I.R. (2004). RPE cells internalize low-density lipoprotein (LDL) and oxidized LDL (oxLDL) in large quantities in vitro and in vivo. Investig. Ophthalmol. Vis. Sci..

[136] Courtois Y. (2010). The role of CD36 receptor in the phagocytosis of oxidized lipids and AMD. Aging.

[137] Rigotti A., Acton S.L., Krieger M. (1995). The class B scavenger receptors SR-BI and CD36 are receptors for anionic phospholipids. J. Biol. Chem..

[138] Martini C., DeNichilo M., King D.P., Cockshell M.P., Ebert B., Dale B., Ebert L.M., Woods A., Bonder C.S. (2021). CD36 promotes vasculogenic mimicry in melanoma by mediating adhesion to the extracellular matrix. BMC Cancer.

[139] Kondo N., Honda S., Kuno S., Negi A. (2009). Positive association of common variants in CD36 with neovascular age-related macular degeneration. Aging.

[140] Honda S., Bessho H., Kondo N., Kusuhara S., Tsukahara Y., Negi A. (2012). Positive association of CD36 gene variants with the visual outcome of photodynamic therapy in polypoidal choroidal vasculopathy. Mol. Vis..

[141] Yanagi Y., Foo V.H.X., Yoshida A. (2019). Asian age-related macular degeneration: From basic science research perspective. Eye (Lond. Engl.).

[142] Bowers C.Y. (1998). Growth hormone-releasing peptide (GHRP). Cell. Mol. Life Sci..

[143] Picard E., Houssier M., Bujold K., Sapieha P., Lubell W., Dorfman A., Racine J., Hardy P., Febbraio M., Lachapelle P. (2010). CD36 plays an important role in the clearance of oxLDL and associated age-dependent sub-retinal deposits. Aging.

[144] Proulx C., Picard É., Boeglin D., Pohankova P., Chemtob S., Ong H., Lubell W.D. (2012). Azapeptide analogues of the growth hormone releasing peptide 6 as cluster of differentiation 36 receptor ligands with reduced affinity for the growth hormone secretagogue receptor 1a. J. Med. Chem..

[145] Cogan D.G., Toussaint D., Kuwabara T. (1961). Retinal vascular patterns. IV. Diabetic retinopathy. Arch. Ophthalmol..

[146] Adamis A.P., Berman A.J. (2008). Immunological mechanisms in the pathogenesis of diabetic retinopathy. Semin. Immunopathol..

[147] Sennlaub F., Valamanesh F., Vazquez-Tello A., El-Asrar A.M., Checchin D., Brault S., Gobeil F., Beauchamp M.H., Mwaikambo B., Courtois Y. (2003). Cyclooxygenase-2 in human and experimental ischemic proliferative retinopathy. Circulation.

[148] Truman J.P., Al Gadban M.M., Smith K.J., Jenkins R.W., Mayroo N., Virella G., Lopes-Virella M.F., Bielawska A., Hannun Y.A., Hammad S.M. (2012). Differential regulation of acid sphingomyelinase in macrophages stimulated with oxidized low-density lipoprotein (LDL) and oxidized LDL immune complexes: Role in phagocytosis and cytokine release. Immunology.

[149] Lopes-Virella M.F., Baker N.L., Hunt K.J., Lyons T.J., Jenkins A.J., Virella G. (2012). High concentrations of AGE-LDL and oxidized LDL in circulating immune complexes are associated with progression of retinopathy in type 1 diabetes. Diabetes Care.

[150] Fu D., Yu J.Y., Wu M., Du M., Chen Y., Abdelsamie S.A., Li Y., Chen J., Boulton M.E., Ma J.X. (2014). Immune complex formation in human diabetic retina enhances toxicity of oxidized LDL towards retinal capillary pericytes. J. Lipid Res..

[151] Abdelsamie S.A., Li Y., Huang Y., Lee M.H., Klein R.L., Virella G., Lopes-Virella M.F. (2011). Oxidized LDL immune complexes stimulate collagen IV production in mesangial cells via Fc gamma receptors I and III. Clin. Immunol..

[152] Ralston J.C., Metherel A.H., Stark K.D., Mutch D.M. (2016). SCD1 mediates the influence of exogenous saturated and monounsaturated fatty acids in adipocytes: Effects on cellular stress, inflammatory markers and fatty acid elongation. J. Nutr. Biochem..

[153] Sasaki M., Kawasaki R., Rogers S., Man R.E., Itakura K., Xie J., Flood V., Tsubota K., Lamoureux E., Wang J.J. (2015). The Associations of Dietary Intake of Polyunsaturated Fatty Acids With Diabetic Retinopathy in Well-Controlled Diabetes. Investig. Ophthalmol. Vis. Sci..

[154] Xu C., Chakravarty K., Kong X., Tuy T.T., Arinze I.J., Bone F., Massillon D. (2007). Several transcription factors are recruited to the glucose-6-phosphatase gene promoter in response to palmitate in rat hepatocytes and H4IIE cells. J. Nutr..

[155] Baranova I.N., Kurlander R., Bocharov A.V., Vishnyakova T.G., Chen Z., Remaley A.T., Csako G., Patterson A.P., Eggerman T.L. (2008). Role of human CD36 in bacterial recognition, phagocytosis, and pathogen-induced JNK-mediated signaling. J. Immunol..

[156] Bamberger M.E., Harris M.E., McDonald D.R., Husemann J., Landreth G.E. (2003). A cell surface receptor complex for fibrillar beta-amyloid mediates microglial activation. J. Neurosci..

[157] Wilkinson K., Boyd J.D., Glicksman M., Moore K.J., El Khoury J. (2011). A high content drug screen identifies ursolic acid as an inhibitor of amyloid beta protein interactions with its receptor CD36. J. Biol. Chem..

[158] Simons E.S., Smith M.A., Dengler-Crish C.M., Crish S.D. (2021). Retinal ganglion cell loss and gliosis in the retinofugal projection following intravitreal exposure to amyloid-beta. Neurobiol. Dis..

[159] Chen H., Herndon M.E., Lawler J. (2000). The cell biology of thrombospondin-1. Matrix Biol..

[160] Tian R., Deng A., Pang X., Chen Y., Gao Y., Liu H., Hu Z. (2022). VR-10 polypeptide interacts with CD36 to induce cell apoptosis and autophagy in choroid-retinal endothelial cells: Identification of VR-10 as putative novel therapeutic agent for choroid neovascularization (CNV) treatment. Peptides.

[161] Upalakalin J.N., Hemo I., Dehio C., Keshet E., Benjamin L.E. (2002). Survival mechanisms of VEGF and PlGF during microvascular remodeling. Cold Spring Harb. Symp. Quant. Biol..

[162] Chu L.Y., Ramakrishnan D.P., Silverstein R.L. (2013). Thrombospondin-1 modulates VEGF signaling via CD36 by recruiting SHP-1 to VEGFR2 complex in microvascular endothelial cells. Blood.

[163] Dong Y., Cai X., Wu Y., Liu Y., Deng L., Chen H. (2017). Insights from Genetic Model Systems of Retinal Degeneration: Role of Epsins in Retinal Angiogenesis and VEGFR2 Signaling. J. Nat. Sci..

[164] Whitcup S.M., Nussenblatt R.B., Lightman S.L., Hollander D.A. (2013). Inflammation in retinal disease. Int. J. Inflamm..

[165] Abe T., Shimamura M., Jackman K., Kurinami H., Anrather J., Zhou P., Iadecola C. (2010). Key role of CD36 in Toll-like receptor 2 signaling in cerebral ischemia. Stroke.

[166] Tannahill G.M., Curtis A.M., Adamik J., Palsson-McDermott E.M., McGettrick A.F., Goel G., Frezza C., Bernard N.J., Kelly B., Foley N.H. (2013). Succinate is an inflammatory signal that induces IL-1β through HIF-1α. Nature.

[167] Lavalette S., Conart J.B., Touhami S., Roubeix C., Houssier M., Augustin S., Raoul W., Combadière C., Febbraio M., Ong H. (2019). CD36 Deficiency Inhibits Retinal Inflammation and Retinal Degeneration in Cx3cr1 Knockout Mice. Front. Immunol..

[168] Nicholas M.P., Mysore N. (2021). Corneal neovascularization. Exp. Eye Res..

[169] Mwaikambo B.R., Yang C., Ong H., Chemtob S., Hardy P. (2008). Emerging roles for the CD36 scavenger receptor as a potential therapeutic target for corneal neovascularization. Endocr. Metab. Immune Disord. Drug Targets.

[170] Ricciuto J., Heimer S.R., Gilmore M.S., Argüeso P. (2008). Cell surface O-glycans limit Staphylococcus aureus adherence to corneal epithelial cells. Infect. Immun..

[171] Mwaikambo B.R., Sennlaub F., Ong H., Chemtob S., Hardy P. (2006). Activation of CD36 inhibits and induces regression of inflammatory corneal neovascularization. Investig. Ophthalmol. Vis. Sci..

[172] Soriano-Romaní L., García-Posadas L., López-García A., Paraoan L., Diebold Y. (2015). Thrombospondin-1 induces differential response in human corneal and conjunctival epithelial cells lines under in vitro inflammatory and apoptotic conditions. Exp. Eye Res..

